# Conservation Agriculture Improves Soil Quality, Crop Yield, and Incomes of Smallholder Farmers in North Western Ghana

**DOI:** 10.3389/fpls.2017.00996

**Published:** 2017-06-21

**Authors:** Jesse B. Naab, George Y. Mahama, Iddrisu Yahaya, P. V. V. Prasad

**Affiliations:** ^1^Savanna Agricultural Research InstituteWa, Ghana; ^2^Department of Agronomy, Kansas State University, ManhattanKS, United States

**Keywords:** conservation agriculture, no-tillage, crop rotation, intercropping, residue retention, soil quality, crop yield, profitability

## Abstract

Conservation agriculture (CA) practices are being widely promoted in many areas in sub-Saharan Africa to recuperate degraded soils and improve ecosystem services. This study examined the effects of three tillage practices [conventional moldboard plowing (CT), hand hoeing (MT) and no-tillage (NT)], and three cropping systems (continuous maize, soybean–maize annual rotation, and soybean/maize intercropping) on soil quality, crop productivity, and profitability in researcher and farmer managed on-farm trials from 2010 to 2013 in northwestern Ghana. In the researcher managed mother trial, the CA practices of NT, residue retention and crop rotation/intercropping maintained higher soil organic carbon, and total soil N compared to conventional tillage practices after 4 years. Soil bulk density was higher under NT than under CT soils in the researcher managed mother trails or farmers managed baby trials after 4 years. In the researcher managed mother trial, there was no significant difference between tillage systems or cropping systems in maize or soybean yields in the first three seasons. In the fourth season, crop rotation had the greatest impact on maize yields with CT maize following soybean increasing yields by 41 and 49% compared to MT and NT maize, respectively. In the farmers’ managed trials, maize yield ranged from 520 to 2700 kg ha^-1^ and 300 to 2000 kg ha^-1^ for CT and NT, respectively, reflecting differences in experience of farmers with NT. Averaged across farmers, CT cropping systems increased maize and soybean yield ranging from 23 to 39% compared with NT cropping systems. Partial budget analysis showed that the cost of producing maize or soybean is 20–29% cheaper with NT systems and gives higher returns to labor compared to CT practice. Benefit-to-cost ratios also show that NT cropping systems are more profitable than CT systems. We conclude that with time, implementation of CA practices involving NT, crop rotation, intercropping of maize and soybean along with crop residue retention presents a win–win scenario due to improved crop yield, increased economic return, and trends of increasing soil fertility. The biggest challenge, however, remains with producing enough biomass and retaining same on the field.

## Introduction

Smallholder farming dominates agriculture in Sub-Saharan African (SSA) operating on less than 2 hectares in total land holding. These are the farmers that supply the urban population with food as well as contribute to the national economies of their individual countries. Yet, smallholder agriculture is constrained by many inter-related factors including low soil fertility, frequent dry spells, drought and unsustainable management practices. Traditional agricultural practices have diminished soil productivity to the extent that many agricultural soils are depleted of nutrients and unable to naturally sustain crop productivity. In the coming decades, a crucial challenge for agriculture in SSA will be meeting food demands without undermining further the environment. Increasing productivity and economic returns to smallholder farming in a sustainable manner is a central challenge to achieving global poverty reduction and environmental management objectives (FAO, 2012a). This challenge can be addressed by identifying, promoting, and realizing widespread and durable adoption of technologies for sustainable agricultural intensification. Conservation agriculture (CA) is one such approach that aims to sustainably improve farm productivity, profits, and food security by combining three principles. These three principles are: minimum mechanical soil disturbance, permanent soil cover, and crop rotation (FAO, 2012b). When these three principles are adhered to, CA is reported to improve soil quality, optimize crop yields and reduce input costs (Hobbs, 2007; Hobbs et al., 2008; Wall, 2008). In North and South America, Australia, and China, several studies have documented the positive effects that conservation practices have in the system, preventing or minimizing soil erosion and soil organic carbon (SOC) loss, improving water capture and use efficiency (Unger, 1990), nutrient cycling and retention and mitigation of GHG emissions (Lal, 2003; Kassam et al., 2009; Palm et al., 2014). Reduced tillage systems in the United States have been shown to reduce soil erosion (Dabney et al., 2004; Wilson et al., 2004), reduce nutrient losses from the field (Kimmell et al., 2001), sequester carbon as a result of increasing organic matter (West and Post, 2002), and increase crop yields (Wilhelm and Wortmann, 2004). In China, Zheng et al. (2014) reported increased crop yield in several locations due to CA. There has been widespread adoption of CA with significant farmer profitability achieved through increased agricultural productivity and reduced input costs.

In Eastern and Southern Africa, considerable research has been done on CA with variable impacts on crop yield reported. In Malawi, no-till and residue retention increased maize yields in two out of five districts after 3 years (Ito et al., 2006). In a 3-year study, CA increased rainwater runoff and negatively impacted cotton yields compared to conventional tillage (CT) (Baudron et al., 2012). Eliminating tillage and retaining residue increased soil water content and crop yields in Kenya, Zambia, and Zimbabwe (Gicheru et al., 2006; Thierfelder and Wall, 2009, 2010; Thierfelder et al., 2012). In Zambia, no-till and residue retention had no impact on continuous maize yields in three out of 4 years (Thierfelder and Wall, 2010). In a 3-year study, TerAvest et al. (2015) reported no significant impact of tillage and residue management on sweet potato, cassava, soybean, or cowpea yields. However CA and CT rotations increased maize yields compared to no-tillage (NT). These studies have given rise to concerns about the ability of CA to transform smallholder agriculture (Gupta and Sayre, 2007; Giller et al., 2009; Farooq et al., 2011). Therefore the impact on crop yield or benefit of CA must be assessed on a case by case basis as the expected effects vary with site-specific conditions, such as soil type, topography, cropping systems, management practices, and climate (Hobbs et al., 2008; Giller et al., 2009; Putte et al., 2010).

In Northern Ghana, continuous cropping and inadequate replacement of nutrients removed in harvested materials, or on site burning of crop residues, and through erosion have hastened soil degradation. Besides low soil fertility, drought, erratic rainfall, and climate change are frequently mentioned by farmers’ as constraints to crop production. One available solution to rebuilding the degraded soils and mitigating the effects of low or erratic rainfall is the development of conservation agricultural practices (CAPs) in intensively managed cropping systems. The goals of such a cropping system must be to increase ecosystem services while simultaneously increasing crop yields and subsequent profitability at the farm level. Improving ecosystem services, with a focus on soil quality, will require the adoption of intensive crop rotations that employ CAPs such as legumes to fix nitrogen, reduced tillage, practices that maintain as much crop residue in the system as possible and integrated nutrient, water and pest management practices. Very little research has been done to illustrate the effects of intensive cropping systems, the use of legumes to fix nitrogen, and the impact of conservation tillage practices on soil quality and crop productivity in Northern Ghana. The objective of this study was to evaluate the short term effects of CAPs that are based on minimum tillage, retention of crop residue, and crop rotation on soil quality, cropping system productivity and profitability of smallholder farmers in Northern Ghana.

## Materials and Methods

### Site Description

The experiments were conducted during the 2010 to 2013 cropping seasons at Nyoli, a farming community located approximately 38 km Southwest of Wa (9°45′ N and 2°30′ W) in the Upper West Region of Ghana. The area has a mono-modal rainfall pattern of about 5–6 months beginning from May to October, with a long term mean annual rainfall of 1026 mm. During the dry season (November–April), the study area is under the influence of the dry south-eastern trade winds (harmattan). The natural vegetation is Guinea Savanna. The major soil type on which agriculture is practiced falls on Ferric lixisols (FAO) or Alfisols (USDA) (Adu and Asiamah, 2003). Soils at the mother and baby trial sites were sandy in texture with low clay contents. Generally all soils were slightly acidic with pH values ranging from 6.0 to 6.4. SOC and total N contents were generally low but comparatively higher in the mother trial site than in farmers’ fields.

### Surveys and Participatory Technology Development Workshops

In order to make technology development and dissemination demand driven and farmer centered, we carried out participatory technology development (PTD) workshops in the community before the start of the study. The purpose of the PTD workshops was to document the community’s livelihoods, major crops cultivated and cropping systems, indigenous CAPs, perceptions of CA, constraints to production, and coping strategies. Details of the baseline survey were published by Dalton et al. (2014). In brief, the main crops cultivated in the area were maize, sorghum, millet, groundnut, and cowpea. Soybean was introduced into the area by an NGO to produce the crop for an oil extraction factory. With regards to cropping pattern, farmers intercrop sorghum or millet with local cowpea, or grow only maize as monoculture. However, during the PTD workshop, farmers chose soybean and maize as the preferred crops to test presumably because soybean is considered as a cash crop and there is ready market for it. Conventional soil preparation involved either hand hoeing or for those who can afford, plowing with bullock-drawn or tractor drawn moldboard plows. In terms of crop residue management, farmers traditionally allow unmanaged livestock grazing during the dry season. The farmers practice with the NGO intervention was land preparation using tractors (moldboard plowing), continuous soybean production with the application of compound fertilizer (N:P:K; 15%N; 15%P_2_O_5_; 15%K_2_O). Harvested soybean plants are typically threshed to obtain the seeds, leaving piles of soybean stems and leaves that are generally burned or simply left to decompose at the threshing site. During one of the PTD workshops, researchers introduced the concept of NT and farmers indicated the things they would like to try. Based on the discussions, “mother and baby trials” were designed and implemented during the 4-year period. The mother trial was researcher managed and had the full complement of treatments while the baby trials had a sub-set of the mother trial treatments.

### Experimental Design

#### Mother Trial

The mother trial was conducted on-farm but was managed by researchers. Treatments were a factorial combination of three tillage methods and three cropping systems. Tillage systems were CT using tractor to plow the land, manual tillage (MT) using hoes (farmer’s practice) and NT. In the NT treatment, after the first rains, annual and perennial weeds were killed by 2.5 l ha^-1^ of glyphosate [*N*-(phosphono-methyl) glycine], applied 10–14 days before planting using a knapsack sprayer followed 3 days later with application of 6 L ha^-1^ of atrazine (14.5% atrazine) [2-chloro-4-ehtylamino-6-isopropylamino-1,3,5-trizine] as a pre-emergence herbicide after planting. Cropping systems were continuous maize, soybean–maize annual rotation and soybean/maize intercropping. The experimental design was split plot with tillage systems as main plot factor and cropping systems as the sub-plot treatment. Treatments were replicated three times. Main plot size was 30 m 30 m and sub-plot size was 30 m × 10 m.

#### Baby Trials

To foster and advance the rapid adoption of CAPs by farmers, 12 farmers’ tested a sub-set of the mother trial treatments in their fields (fully managed by farmers). The baby trials treatments were a factorial combination of two tillage systems (conventional and no-till), and two cropping systems (continuous maize cropping and soybean–maize annual rotation). In the CT treatment, the plots were disk plowed using tractor while in the NT plots glyphosate was applied to kill all vegetation before sowing into the trash using cutlasses. The experimental design was randomized complete block with farmers as replicates. Each treatment plot size was 50 m × 20 m.

### Soil Sampling and Analysis

Initial soil samples were collected in 2010 at the 0–0.15, 0.15–0.30, and 0–0.30 m soil depth of each treatment plot of the mother trial and farmers’ fields (baby trials) for determination of texture, soil pH, SOC, total soil nitrogen (TSN) and mineral nitrogen prior to the establishment of the experiments. In May 2014, soil samples were taken again for SOC, TSN, and mineral N contents analyses at the soil depth intervals of 0–0.15 and 0.15–0.30 m. Using a soil auger, 10 cores per plot were taken and bulked to make a composite sample. The samples were air dried and a portion used for the determination of soil texture. The remaining soil samples were ground and sieved to 2 mm for determination of pH, SOC, and TSN. Particle size distribution was determined by the hydrometer method. Soil bulk density was measured in 2010 and 2014 by taking soil samples from the 0–0.10 m soil depth using metal core samplers of known weight and volume. Soil bulk density was determined by taking undisturbed soil cores, oven drying at 105°C for 48 h. Bulk density was calculated as mass of oven dry soil core divided by volume of the core (Blake and Hartage, 1986). Soil pH was measured in 1:2 soil/water solution using a glass electrode. SOC was determined by wet oxidation with potassium dichromate and sulfuric acid procedure (Walkley and Black, 1934) as outlined by Nelson and Sommers (1982). Total soil N was determined by the micro-Kjeldahl procedure as described by Anderson and Ingram (1993). Mineral N content of the soil was analyzed by the rapid steam distillation method (Bremner and Keeney, 1966).

### Trial Management

The mother trial was managed by researchers with the support of the local farmers in carrying out operations such as sowing, weeding, fertilizer application and harvesting. The baby trials were managed by farmers themselves with technical support from researchers and the village extension agent. In both mother and baby trials, improved maize (Zea *mays* L.) variety cv. *Obatanpa*, and soybean (*Glycine max* L. Merril) variety cv. *Jenguma*, were used each year. Maize seed was sown in rows 0.75 m apart while soybean was sown in rows spaced 0.50-m. In 2010, farmers used cutlasses to make holes within the row before sowing. In subsequent years, the farmers recommended the fabrication of a 7-shape metal hoe which was used to cut slits in the rows in the no-till plots before sowing. In 2011 and subsequent cropping seasons, the farmers recommended a novel way to overcome the initial slow growth after sowing. Maize seeds were pre-germinated and transplanted 2–3 weeks after emergence usually after a significant rainfall. Weeds in the CT and MT plots were controlled by hand hoeing while in the no-till plots weed control was achieved through the *in situ* application of a mixture of 2.5 L ha^-1^ glyphosate and 6 L ha^-1^ of atrazine as a pre-emergence herbicide after planting. If weeds reappeared after the herbicide application, they were removed by hand pulling. Each year, compound fertilizer (N–P–K; 15% N, 15% P_2_O_5_, and 15% K_2_O), was applied at a rate of 64 N, 16 P, and 31 K kg ha^-1^ to the maize either sole cropped or in the intercrop. This was supplied by applying 250 kg ha^-1^ of N–P–K at seeding and 125 kg ha^-1^ of ammonium sulfate (21% N) approximately 3 weeks after planting. No fertilizer was applied to the soybean either sole or intercropped with maize in both trials.

At final harvest each year in the mother trial, maize ears from the middle four rows (90 m^2^) of each plot were harvested for grain yield assessment. For intercrops, the inner two rows (45 m^2^) of each crop were harvested for grain yield assessment. Aboveground maize crop residue was estimated by cutting and weighing all the stover from each plot after all maize ears were removed. A sub-sample of plants was taken weighed and oven dried at 70°C until constant weight. Sample dry weights were used to convert total maize stover from each plot to dry matter on an area basis. The remaining maize stover was left on the plots as mulch after weighing. For soybeans, all the plants were pulled off by hand, placed on a tarpaulin and threshed and grain yield determined. In the mother trial, the stover was uniformly spread on the soil surface of each plot. After threshing of soybean on tarpaulins, the residue was returned to the respective plots. In the baby trials, maize ears from the middle four rows (150 m^2^) of each treatment plot were harvested for grain yield assessment. The ears were air dried, shelled, and the grain weighed. For soybean, plants from the whole plot (500 m^2^) were harvested, air dried on bare ground, threshed manually and the grain weighed. Aboveground maize crop residue was assessed on five farmers’ fields as described for the mother trial. Farmers’ were encouraged to also leave the maize crop residues on the plots. Soybean crop residue could not be assessed because of the method of harvesting and processing for grain. Daily rainfall was measured with a rainfall gauge installed in the village.

### Cost-to-Benefit Analysis

Costs and benefits of each CA practice were compared using partial budgeting which included only costs and benefits. The costs included land preparation either by tractor or herbicide, fertilizers, and labor costs for sowing, weeding, application of herbicide and fertilizer. The gross margin (*GM*) or profit was computed for each CA practice as follows:

$$\begin{array}{c} GM=Y\times P-TV \end{array}$$

where *Y* is grain yield of maize or soybean (kg ha^-1^), *P* is the selling price of the grain at harvest, and *TVC* is the total variable cost or costs related to the CA practice in US$ ha^-1^. Price of maize grain at harvest was the average of the market price during harvest (October to January) from 2010 to 2013. The price of soybean was the average of the price offered by the NGO from 2010 to 2013. Costs of maize and soybean seed, herbicide, and fertilizers were as purchased from input dealers each year in the area. Labor was valued at the wage rate of hired farm laborers during the cropping season. Benefit-cost analysis (i.e., the ratio between the added benefits and the added costs) was used to determine the profitability or otherwise of the different CA practice. All monetary values were converted to US dollars (USD) at the mean exchange rate of the Ghana Cedis during the field experiments (2010: 0.688 GHC = 1 US$; 2011: 0.646 GHC = 1 US$; 2012: 0.520 GHC = 1 US$; 2013: 0.458 GHC = 1US$).

### Statistical Analysis

All agronomic data from the mother trial were analyzed using a two factor (tillage and cropping systems) split-plot design with Duncan’s Multiple Range Test (DMRT) at 5% level of significance for separation of means. Agronomic data from the baby trials (farmers’ fields) were analyzed as two factors randomized complete block design with 12 farmers’ fields as replicates. Soil carbon, total nitrogen, and mineral nitrogen data from both mother and baby trials were analyzed as three factors (tillage, cropping system, and soil depth) randomized complete block designs. All ANOVA was done in SigmaPlot 11.0 statistical package. All data passed the normality and homogeneity of variances test (see Supplementary Material Data Sheet 1).

## Results

### Rainfall during the Study

**FIGURE 1:**
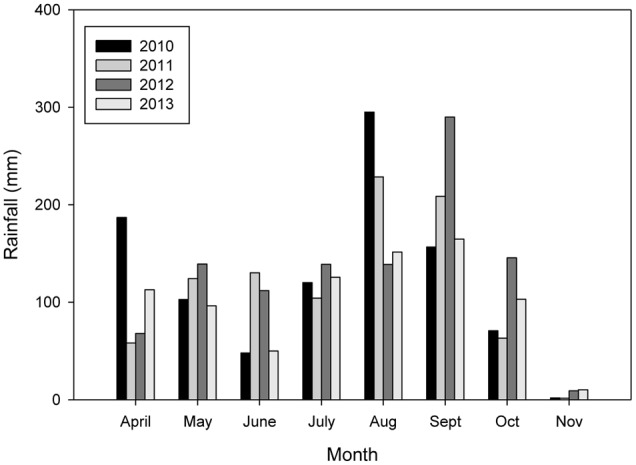
Rainfall distribution during the growing season from 2010 to 2013.

Total rainfall and its distribution within the season varied from year-to-year (**Figure 1**). The highest total rainfall was measured during the 2012 season (1041.5 mm) and the lowest was measured during the 2013 season (814 mm). Total rainfall was 981 and 918 mm in 2010 and 2011, respectively. Rainfall distribution was comparatively better in 2012 and 2013 with rains continuing into October and November than in other years. June was comparatively drier in 2010 (total rainfall = 48 mm) and 2013 (total rainfall = 50 mm) than in 2011 (total rainfall = 130 mm) and 2012 (total rainfall = 112 mm). This delayed planting of the trials 2010 and 2013 until about mid-July. The long dry spells were followed by frequent heavy rains in August in 2010 (total rainfall = 295 mm) and 2011 (total rainfall = 229 mm) which caused waterlogging in some farmers fields.

### Mother Trial

#### Tillage and Cropping System Effects on Soil Bulk Density

Measurements made in 2014 showed significant differences between tillage systems in bulk density in the mother trial. NT increased bulk density compared to MT and CT practices (**Table 1**). There was no cropping systems influence on bulk density.

**Table 1:** Soil bulk density (g cm^-3^) as affected by tillage and cropping system in 2014.

		Cropping system			
Experiment	Tillage system	CMZ	SB–MZ	SB/MZ	Mean
Mother trial site	CT	1.50a	1.52a	1.45a	1.49A
	MT	1.62a	1.65a	1.51a	1.59AB
	NT	1.66a	1.79a	1.68a	1.71B
	Mean	1.59a	1.65a	1.54a	
Baby trials (*n* = 5)	CT	1.56a	1.50a		1.53A
	NT	1.69a	1.66a		1.68B
	Mean	1.63a	1.58a		

*CT, conventional tillage; MT, manual tillage; NT, no-tillage; CMZ, continuous maize;*

*SB–MZ, soybean–maize rotation; SB/MZ, soybean/maize intercrops.*

*Means within a column followed by similar upper case letters are not significantly different.*

*Means within a row followed by similar lower case letters are not significantly different.*

#### Tillage and Cropping System Effects on Soil Carbon and Nitrogen Contents

In the mother trial, there was a significant (*P* = 0.032) interaction of tillage and cropping system on SOC content measured in 2014. Within tillage systems, CT with sole cropping of maize decreased SOC compared with soybean–maize annual rotation and soybean/maize intercropping (**Table 2**). MT or NT with soybean–maize rotation maintained higher SOC content compared to continuous sole cropping or intercropping (**Table 2**). Within cropping systems, MT or NT maintained higher SOC with continuous sole cropping but no differences between tillage systems with soybean–maize annual rotation. NT soybean/maize intercropping maintained higher SOC compared with CT (**Table 2**).

**Table 2:** Soil organic carbon, total nitrogen, and mineral nitrogen averaged across soil layers, as affected by tillage and cropping system in 2014 in the mother trial.

	Cropping system			
Tillage system	Sole maize	SB–MZ	SB/MZ	Mean
**Organic carbon (g kg^-1^)**				
CT	3.42aA	4.26bA	4.82acA	4.17A
MT	4.14aB	5.29aA	4.52aAB	4.65A
NT	5.40aAB	5.39bA	5.20bcB	5.33B
Mean	4.32a	4.98b	4.85b	
**Total nitrogen (g kg^-1^)**				
CT	0.37aA	0.41bA	0.43bA	0.40A
MT	0.54aA	0.62bA	0.64bA	0.60B
NT	0.59aA	0.71bA	0.66bA	0.65B
Mean	0.50a	0.58b	0.58b	
**Mineral nitrogen (mg kg^-1^)**				
CT	20.3aA	26.6bA	24.0bA	23.6A
MT	40.5acA	33.8bB	32.2cA	35.5B
NT	35.1aA	51.1bcB	47.4cA	44.5C
Mean	31.9a	37.2a	34.5a	

*CT, conventional tillage; MT, manual tillage; NT, no-tillage; CMZ, continuous maize;*

*SB–MZ, soybean–maize rotation; SB/MZ, soybean/maize intercrops.*

*Means within a column followed by similar upper case letters are not significantly different.*

*Means within a row followed by similar lower case letters are not significantly different.*

Total soil N content followed a similar trend as SOC. There were significant main effects of tillage and cropping system on TSN (**Table 2**). Averaged across cropping systems, CT decreased TSN after 4 years compared to MT and NT systems (**Table 2**). Soybean–maize annual rotation and intercropping maintained higher TSN than sole maize cropping, when averaged across tillage systems.

There was significant (*P* = 0.006) interaction of tillage and cropping system on mineral nitrogen measured at the beginning of the cropping season in 2014. Mineral N contents were higher with MT and NT systems compared with CT in the soybean–maize rotation system (**Table 2**). Tillage system did not influence mineral N content in the sole and intercropping systems. Within tillage systems, mineral N content were higher with soybean–maize rotation and intercropping than sole maize cropping (**Table 2**).

#### Annual Crop Residue Production

**FIGURE 2:**
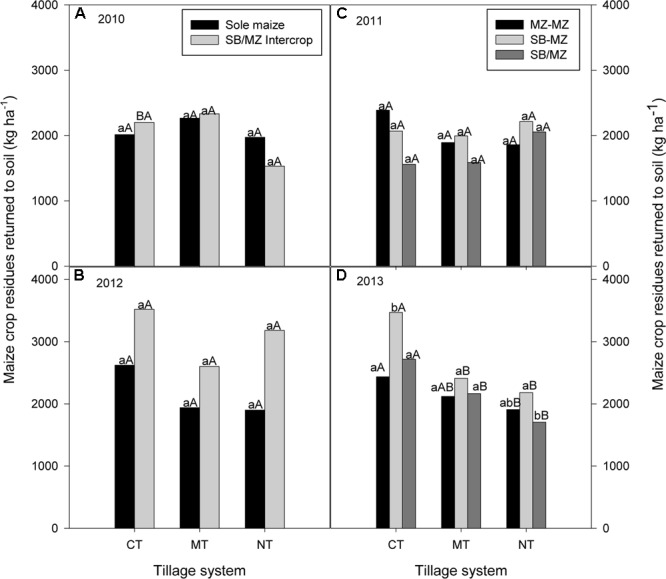
Tillage and cropping system effects on maize crop residues returned to the soil in **(A)** 2010, **(B)** 2012, **(C)** 2011, and **(D)** 2013 in the mother trial.

There was no significant differences between tillage or cropping systems on the amount of crop residues produced and returned to the soil in 2010 (**Figure 2A**). However, highest total crop residue was obtained in MT (intercropping = 2333 kg ha^-1^; sole maize = 2266 kg ha^-1^) followed by CT (intercropping = 2200 kg ha^-1^; sole maize = 2014 kg ha^-1^) and lowest in NT (intercropping = 1532 kg ha^-1^; sole maize = 1973 kg ha^-1^). Similarly, there were not significant differences in maize crop residue production due to tillage or cropping system in 2011 (**Figure 2C**). However, highest crop residues was produced in CT (range = 1560 to 2387 kg ha^-1^) followed by NT (range = 1860 to 2214 kg ha^-1^) and lowest in MT (range = 1587 to 1994 kg ha^-1^). In 2012, there was no significant interaction between tillage and cropping systems on crop residue production (**Figure 2B**). Total crop residues were highest in intercropping for all tillage systems (range = 2600 to 3521 kg ha^-1^) compared with sole cropping (range = 1901 to 2621 kg ha^-1^) (**Figure 2B**). However, averaged across tillage systems, intercropping produced significantly higher maize crop residue (3100 kg ha^-1^) than sole maize (2154 kg ha^-1^) that was returned to the soil in 2012. In 2013, there were significant effects of tillage and cropping system on maize crop residue production (**Figure 2D**). Total biomass were highest in maize following soybean (range = 2181 to 3468 kg ha^-1^) followed by intercropping (range = 1706 to 2718 kg ha^-1^) and lowest in sole maize (range = 1908 to 2436 kg ha^-1^) for all tillage systems. Averaged across cropping systems, CT produced higher crop residue than MT and NT. CT maize following soybean produced significantly higher crop residues than sole maize and maize intercropped with soybean when averaged across tillage systems (**Figure 2D**).

#### Maize and Soybean Grain Yields

**FIGURE 3:**
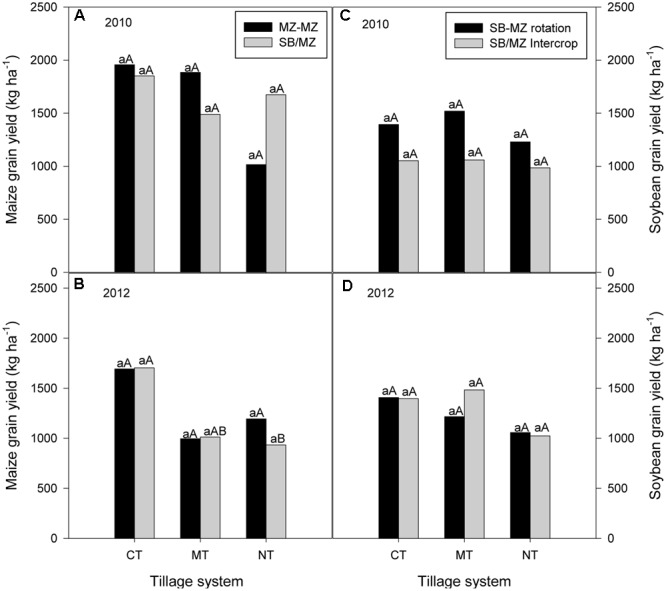
Tillage and cropping system effects on grain yields of maize **(A,B)** and soybean **(C,D)** during the 2010 and 2012 cropping seasons in the mother trial.

In 2010, which was the first year of the experiment, there was no rotation effect and so results are presented for maize grain yield of sole maize and soybean/maize intercrop and soybean grain yield from sole soybean and soybean/maize intercrop. As with crop residue production, there was no significant interaction of tillage and cropping system on maize grain yield (**Figure 3A**) in 2010. In 2012, CT soybean/maize intercropping produced higher maize grain yield than MT and NT soybean/maize intercropping (**Figure 3B**). Similarly, there were no significant differences between tillage or cropping systems or their interaction on soybean grain yields in 2010 and 2012 (**Figures 3C,D**).

**FIGURE 4:**
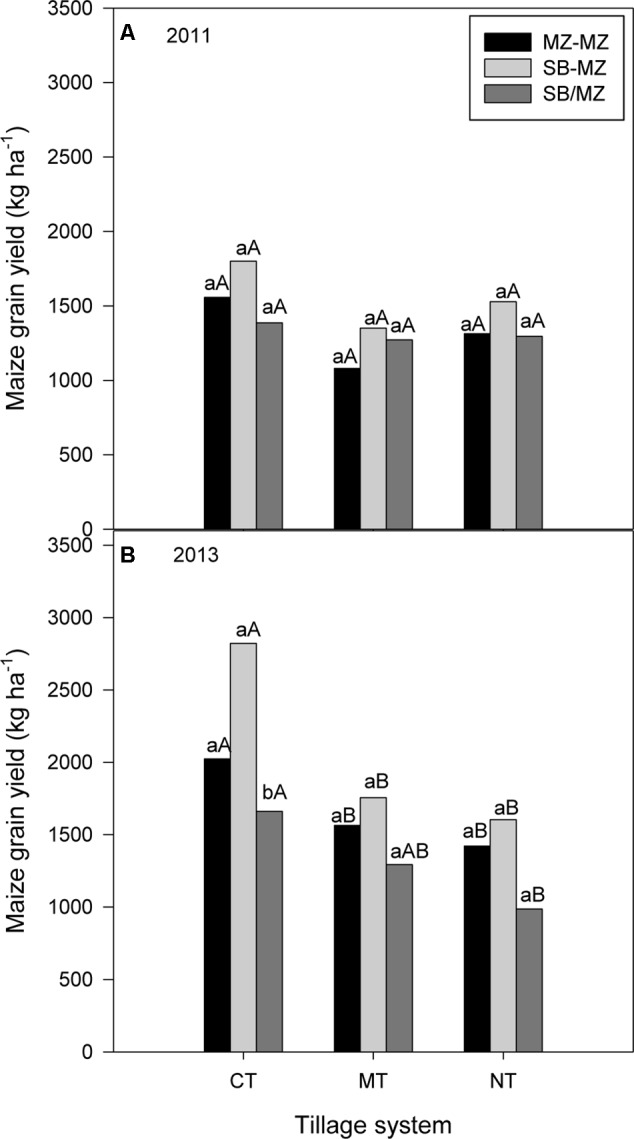
Tillage and cropping system effect on maize grain yield in **(A)** 2011 and **(B)** 2013 cropping seasons in the mother trial.

In 2011, there were no differences between tillage or cropping systems or their interaction on maize grain yield although maize following soybean tended to be higher than sole or intercropped maize (**Figure 4A**). However in 2013, there was a significant interaction (*P* = 0.019) between tillage and cropping system on maize grain yield (**Figure 4B**). Within tillage systems, CT sole maize and maize following soybean grain yield were significantly higher than maize intercropped with soybean grain yield. There were no difference between cropping systems in maize grain yield under MT and NT. Within cropping systems, sole maize and maize following soybean grain yields were significantly higher under CT than under manual and no-till (**Figure 4B**). Also, CT intercropping gave higher maize grain yield than NT intercropping.

### Baby Trials

#### Tillage and Cropping Systems Effects on Soil Bulk Density, Carbon, and N Contents

Averaged across cropping systems, NT resulted in significantly higher bulk density compared to CT (**Table 1**). Averaged across farmers’ fields, there was no significant influence of tillage or cropping system or the interaction, on SOC, TSN, and mineral N contents after 4 years (**Table 3**).

**Table 3:** Soil organic carbon, total nitrogen, and mineral nitrogen contents averaged over farmers’ fields in 2014.

	Cropping system		
Tillage system	CMZ	SB–MZ	Mean
**Organic carbon (g kg^-1^)**			
CT	3.68aA	4.02aA	3.85A
NT	4.04aA	3.90aA	3.97A
Mean	3.86a	3.96a	
**Total nitrogen (g kg^-1^)**			
CT	0.33aA	0.36aA	0.34A
NT	0.36aA	0.35aA	0.35A
Mean	0.34a	0.35a	
**Mineral nitrogen (mg kg^-1^)**			
CT	20.6aA	23.0aA	21.8A
NT	20.3aA	23.1aA	21.7A
Mean	20.5a	23.0a	

*CT, conventional tillage; NT, no-tillage; CMZ, continuous maize; SB–MZ,*

*soybean–maize rotation.*

*Means within a column followed by similar upper case letters are not significantly different.*

*Means within a row followed by similar lower case letters are not significantly different.*

#### Crop Residue Production

**FIGURE 5:**
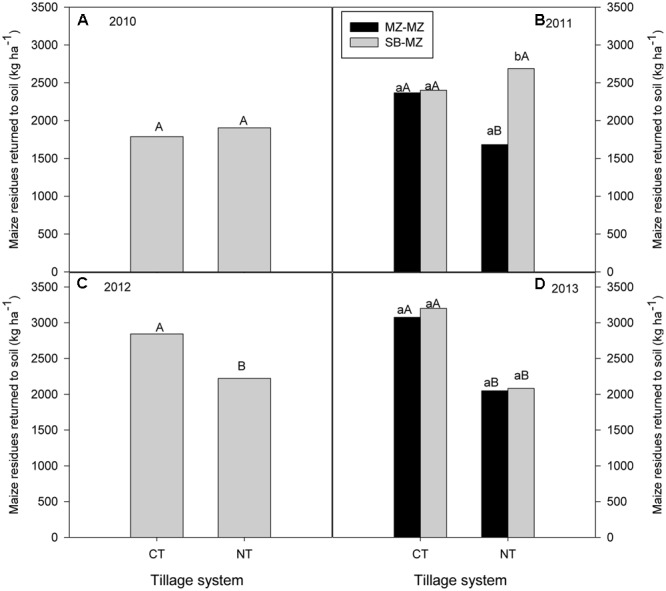
Tillage and cropping systems effect on amount of maize crop residues produced and returned to the soil in the baby trials during **(A)** 2010 **(B)** 2011 **(C)** 2012 and **(D)** 2013 cropping seasons.

In 2010, sole maize crop residue produced ranged from 520 to 2180 kg ha^-1^ in CT plots and from 580 to 2240 kg ha^-1^ in NT plots. Averaged for all farms, there was no significant difference between tillage systems in maize crop residue production (**Figure 5A**). In 2011, there was no difference between tillage or cropping systems in maize crop residue production although NT maize following soybean had high crop residue production (**Figure 5B**). Crop residue of maize following soybean were higher (CT = 2402 kg ha^-1^; NT = 2688 kg ha^-1^) than continuous maize crop residue (CT = 2367 kg ha^-1^; NT = 1683 kg ha^-1^). In 2012, sole maize crop residue was higher in CT plots (range = 1570 to 2255 kg ha^-1^) than in NT plots (range = 1115 to 1849 kg ha^-1^). Averaged across farms, the amount of crop residue produced was significantly higher with CT than with NT (**Figure 5C**). In 2013, CT sole maize and maize following soybean produced significantly higher crop residues than NT sole maize and maize following soybean (**Figure 5D**). Within tillage system, there was no difference between cropping systems (CT: soybean–maize rotation = 3201 kg ha^-1^; continuous maize = 3075 kg ha^-1^; NT: soybean–maize = 2082 kg ha^-1^; continuous maize = 2049 kg ha^-1^) in crop residue of maize produced.

#### Maize and Soybean Grain Yields

**FIGURE 6:**
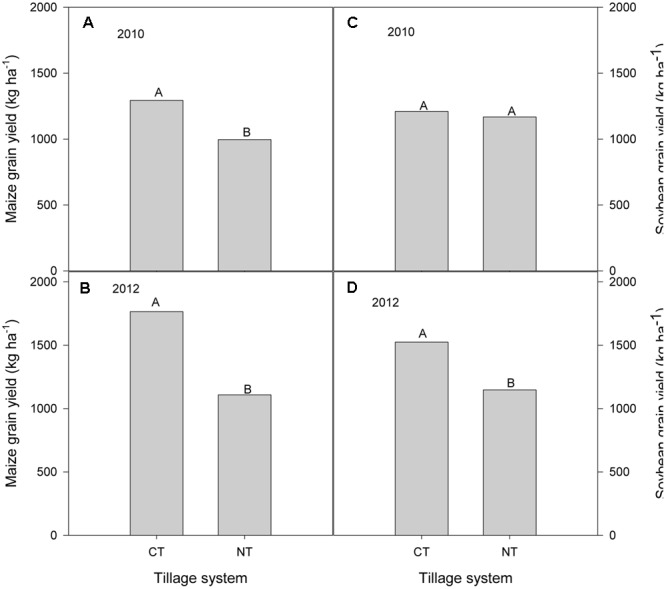
Tillage effects on sole maize **(A,B)** and sole soybean **(C,D)** grain yields in the baby trials during the 2010 and 2012 cropping seasons.

As expected in on-farm trials, there was wide range in maize and soybean grain yield. In 2010 season, maize grain yields on farmers’ fields ranged from 520 to 2700 kg ha^-1^ under CT and from 400 to 1820 kg ha^-1^ with NT. Averaged across farms, CT produced significantly higher maize grain yields compared to NT, translating to 23% more yield than in NT (**Figure 6A**). In 2012 season, maize grain yields ranged from 853 to 2733 kg ha^-1^ under CT and from 333 to 2173 kg ha^-1^ with NT. Averaged across farms, CT produced 37% more maize grain yield than NT (**Figure 6B**).

Soybean grain yields ranged from 180 to 2257 kg ha^-1^ and from 298 to 3000 kg ha^-1^ with CT and NT, respectively, in 2010 cropping season. There was no difference between tillage systems in soybean grain yields when averaged for all farms (**Figure 6C**) but in 2012, CT significantly increased soybean grain yields by 25% compared with NT (**Figure 6D**) when averaged for all farms.

**FIGURE 7:**
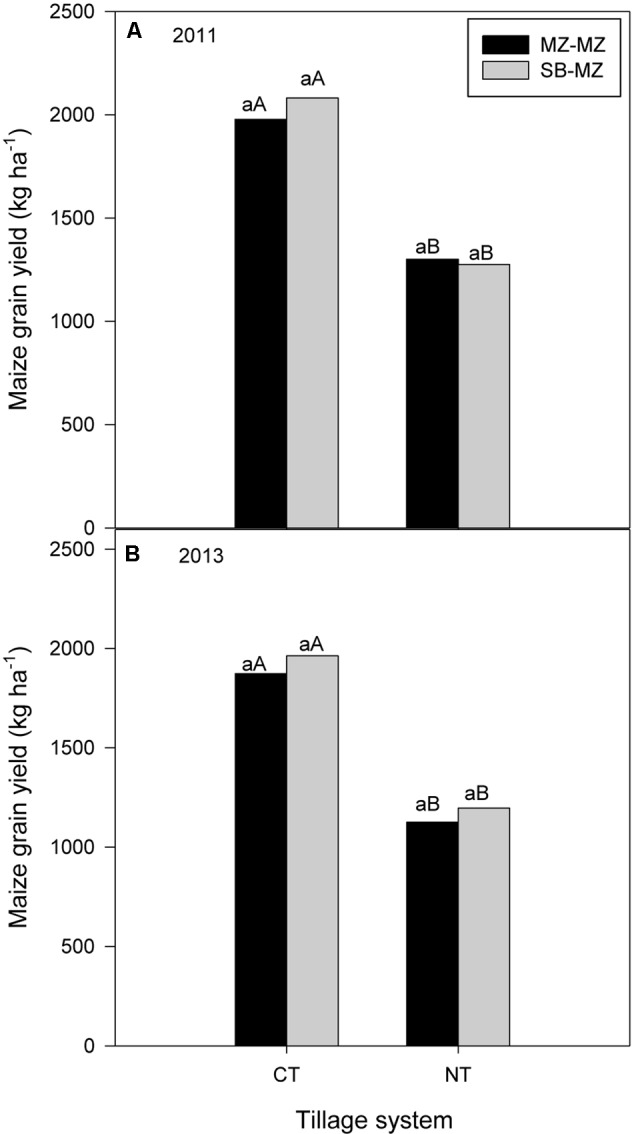
Tillage and cropping system effect on maize grain yield in the baby trials during **(A)** 2011 and **(B)** 2013 cropping seasons.

In 2011 and 2013 seasons, averaged across farms, CT produced significantly higher maize grain yield compared to NT (**Figures 7A,B**). Within tillage systems, there were no differences between cropping systems, although maize following soybean tended to have higher absolute values.

#### Cost-Benefit Analysis for Baby Trials

Cost-benefit analyses averaged across farmers’ fields for each year are given in **Table 4**. In 2010, the average cost of producing maize per hectare was estimated as $379 and $301 for CT and NT, respectively. This represents a 21% reduction in cost of production with NT compared to CT. The gross margin with CT is 34% higher than with NT. However, the benefit-to-cost ratio for both CT and NT averages about 0.2. Returns to labor is, however, high for CT and NT.

**Table 4:** Comparison of conventional and no-tillage cost and benefits for smallholder farmers’ maize and soybean production from 2010 to 2013 cropping seasons in Nyoli, Ghana.

	Maize		Soybean–maize	
	monocropping		annual rotation	
Costs	CT	NT	CT	NT
**2010**				
Labor	60.54	58.48	112.69	112.69
Purchased inputs	318.54	242.18	103.20	4059
Total variable cost	379.09	300.66	215.89	153.29
Revenue (US$)	462.94	356.33	432.89	417.86
Gross margin (US$)	83.85	55.67	217.00	264.58
Benefit/cost ratio	0.22	0.19	1.01	1.73
Returns to labor	7.65	6.09	3.84	3.71
Labor productivity	21.57	16.60	20.17	19.47
**2011**				
Labor	59.43	59.43	116.28	116.28
Purchased inputs	312.02	229.98	109.82	51.03
Total variable cost	371.45	289.41	226.10	167.31
Revenue (US$)	689.66	445.09	739.38	453.01
Gross margin (US$)	318.21	155.69	513.28	285.69
Benefit/cost ratio	0.86	0.54	2.27	1.71
Returns to labor	11.60	7.49	6.36	3.90
Labor productivity	32.95	21.67	34.68	21.25
**2012**				
Labor	66.56	66.04	105.98	105.98
Purchased inputs	261.56	203.84	98.80	41.08
Total variable cost	328.12	269.88	204.78	147.06
Revenue (US$)	477.26	299.60	412.09	310.42
Gross margin (US$)	149.14	29.72	207.31	163.36
Benefit/cost ratio	0.45	0.11	1.01	1.11
Returns to labor	7.17	4.54	3.89	2.93
Labor productivity	29.42	18.47	25.40	19.13
**2013**				
Labor	72.36	68.24	102.50	93.24
Purchased inputs	463.00	178.62	96.18	45.34
Total variable cost	535.36	246.86	198.68	138.68
Revenue (US$)	557.89	335.21	360.22	347.71
Gross margin (US$)	22.53	88.35	161.54	209.03
Benefit/cost ratio	0.04	0.36	0.81	1.51
Returns to labor	7.71	4.91	3.51	3.73
Labor productivity	31.23	18.77	20.17	19.47

In 2010, the average variable cost per hectare of producing soybeans was estimated as $216 and $153 for CT and NT, respectively. This implies a reduction in cost of production per hectare of about 29%. The gross margins per hectare for CT and NT are, respectively, estimated to be $217 and $265. The results show an increase of about 22% in gross margin for NT compared to CT. The benefit-cost ratio per hectare for CT and NT are about 1.00 and 1.7, respectively. This implies NT under farmers’ condition is more profitable for soybeans than CT. the returns to labor for both CT and NT are higher with labor productivity also higher for both technologies.

In 2011, total variable costs of continuous maize production per hectare were estimated to be $371 and $289 for CT and NT, respectively. This represents a production cost reduction of 22% for NT compared to CT. The gross margin for continuous maize production with CT is also higher than with NT. However, the benefit cost ratio for both CT and NT are less than unity. Total variable costs of production of maize following soybean in rotation in 2011 were estimated to be $226 and $167 for CT and NT, respectively. This represents a 26% reduction in cost of production per hectare for NT. Gross margins are estimated to be $513 and $286 per hectare for CT and NT, respectively. The benefit-cost ratios for CT and NT are both greater than 1. The results also show higher returns to labor and labor productivity.

In 2012 cropping season, the average costs of production per hectare of maize were estimated to be about $328 and $270 for CT and NT, respectively. This represents about 18% reduction in cost of production with NT. The gross margin for CT is, however, 80% higher than with NT. The benefit-cost ratio for CT and NT are, however, less than 1. Returns to labor and labor productivity are, however, high for the tillage systems. The total variable cost of production per hectare of soybean with CT and NT were estimated to be about $205 and $147, respectively. This represents a 28% reduction in cost of production per hectare for NT. Gross margins on the other hand were 21% higher with CT compared to NT. The benefit-cost ratio for both CT and NT are slightly greater than 1.

In 2013, total variable costs of continuous maize production per hectare with CT and NT were estimated to be $535 and $247, respectively. The reduction per hectare is about 53% for NT. The gross margins for CT and NT per hectare were estimated to be $22 and $88, respectively. This represents a 200% increase in gross margins for NT compared to CT. However, the benefit-cost ratios for both tillage systems are less than 1. Total variable costs of maize following soybean production were estimated to be $199 and $139, respectively, for CT and NT. This represents a 30% reduction in cost of production per hectare for NT compared to CT. The benefit-cost ratio for NT is greater than 1 but less than 1 for CT.

## Discussion

### Tillage and Cropping System Effects on Crop Residue Production and Retention

Retention of crop residue on the soil surface as mulch is an essential component of CA intended to increase carbon inputs and enhance ecosystems benefits such as soil fertility, improved soil water relations, and biological properties (Palm et al., 2014). The level of benefits derived is, however, dependent on the amount of residue retained on the field as well as other environmental conditions. Crop residue retained as surface mulch in the range 2–6 Mg ha^-1^ has been reported to increase infiltration, soil moisture, and boost crop yields (Derpsch, 1988; Gicheru et al., 2006; Mupangwa et al., 2007, 2012). Other studies (Ghimire et al., 2012; Paul et al., 2013) found no differences in soil C concentration between CT and RT when both tillage systems received 4.0 Mg ha^-1^ of residue 4 and 6 years, respectively, after application. In this study, annual crop residue returned to the soil surface in the researcher managed mother trial ranged from 2 to 3.5 Mg ha^-1^. In farmers’ fields, annual crop residue produced ranged from 1.0 to 3.0 Mg ha^-1^. The low amounts of crop residue production may be due to the low fertility status of the soils in this area coupled with inadequate fertilizer application. In many cropping systems in tropical Africa where the soils are severely deficient in nitrogen and phosphorus, very little crop residues are available for mulching due to overall low biomass production levels, and residue removal by livestock particularly during the dry season when communal grazing is common. Achieving adequate soil cover in CA may be difficult for smallholder farmers in this community using only *in situ* biomass production. Application of adequate mineral fertilizer is a prerequisite for greater crop residue production that may allow sufficient residues to be returned to fields and some to be removed for other uses without detrimental effects to the soil and subsequent crop yields. Farmers may also be able to compensate for low biomass production by intercropping agroforestry trees or relay cropping with grain legumes such as pigeon pea.

### Tillage and Cropping Systems Effects on Crop Yields

In the researcher managed mother trial, our results show that there were no significant differences between the CA practices of MT and NT and CT in maize grain yield in the first three seasons although CT tended to have higher absolute values. Averaged across cropping systems, CT increased maize grain yield by 11 and 29% in 2010, 22 and 15% in 2011 season, and 41 and 37% in 2012 season compared to MT and NT, respectively, in each year. In 2013, CT had the largest impact on maize yields, increasing yields over MT and NT by 41 and 49%. These results agree with previous studies in southern and eastern Africa which found either no yield benefits of CA over CT in the initial years (Thierfelder and Wall, 2010; Thierfelder et al., 2013) or that CT significantly increased maize yield compared to NT (TerAvest et al., 2015). Our results are also similar to Ngwira et al. (2012, 2013) who observed no significant differences in maize yield during the first four cropping seasons in a high rainfall area but contrast the results in drier areas where yield benefits of CA over conventional practices were recorded from the first cropping season. Good management is the first step for detecting tillage effects (Baudron et al., 2012). The comparatively good yields under MT and NT may be due to an effective herbicide regime coupled with early weed control, early fertilizer application to overcome possible N immobilization as suggested by other authors (Giller et al., 2009) and better moisture regime with residue retention.

Cropping system had no significant impact on maize grain yield in all years although maize following soybean tended to have higher yields. The yield advantages of crop rotation over continuous maize cropping were 243, 270, and 215 kg ha^-1^ in 2011 while in 2013 season, yield advantages were 431, 192, 182 kg ha^-1^ for CT, MT, and NT, respectively, in each year. The small but insignificant yield advantage of crop rotation may be attributed to fixation of atmospheric N and other rotation effects. Inclusion of legumes in a rotation, either sole-cropped or intercropped, can increase maize yields, soil N and fertilizer-use efficiency (Snapp et al., 2010). In addition to increasing soil N, crop rotations provide many ecosystem services that interact to boost crop yields, including breaking up pest and disease cycles, minimizing weeds, increasing biodiversity and recycling nutrients (Kassam et al., 2009).

In contrast to the researcher managed mother trial, CT had the greatest impact on maize and soybean yields in the farmer managed baby trials irrespective of cropping system, increasing yields over NT in all 4 years. Crop rotation had no effect on yields irrespective of the tillage system. There were small but insignificant yield advantages of crop rotation over monocropping with CT but was not consistent with NT. The lower yields with no-till in farmers’ fields was largely due to lack of experience by some farmers in the initial years and ineffective herbicide application leading to competition from weeds. Additionally, no-till systems without adequate residue retention can decrease crop yields compared to CT systems (Thierfelder et al., 2013). The lack of significant influence of crop rotation on maize yield may be due to the removal of whole soybean plants from the field for threshing elsewhere and not returning the residue. A legume only adds significant nitrogen to the soil for the benefit of the following crop if the entire biomass (stems, leaves, roots) is incorporated into the soil.

### Tillage and Cropping System Effects on Soil Quality

Conservation agriculture influences soil physical properties such as bulk density and porosity as well as chemical and biological properties (Verhulst et al., 2010). In this study, bulk density was higher under NT soil than under CT soils in the mother and baby trials after 4 years. The higher bulk density under NT may be due to raindrop impact in these sandy soils whereas CT cause more disturbances and mixing than NT and thus reduces bulk density. Our results are in agreement with Yang and Wander (1999) and Gal et al. (2007) but in contrast with Angers et al. (1997) and Ussiri and Lal (2009) who reported lower soil bulk densities under conservation systems relative to tilled plots. The experiment was established on land that had been under fallow (native vegetation regrowth) for the past 10 years. Initial SOC and TSN values at the start of the experiment in 2010 were on average 7.0 g kg^-1^ and 0.8 g kg^-1^, respectively. In 2014, SOC generally decreased by 38, 34, and 24% while TSN decreased by 50, 25, and 7% with CT, MT, and NT treatments, respectively, when averaged across cropping systems. Several studies in Africa have reported similar SOC and TSN declines within 2–6 years following the conversion from native to cultivated land (Brams, 1971; Ayanaba et al., 1976; Juo and Lal, 1979; Solomon et al., 2000). Despite the general decline in SOC and TSN with time, results from our research show that minimum and NT systems maintained higher SOC and total N than CT. These results agree with Lal (1976), Agboola (1981), and Muchabi et al. (2014). Higher SOC and total N have been reported in CA systems with crop residue retained as surface mulch than conventional tilled systems with residue incorporated in long-term experiments in Mexico (Govaerts et al., 2006; Verhulst et al., 2011). In this study, the amount of crop residue returned to the soil annually was similar for all tillage and cropping systems. Crop residues were incorporated into the soil during plowing in the CT plots, partially in the MT plots and spread on the soil surface in the NT plots and protected from animals. The higher SOC and total N with MT and NT in this study can be attributed to the limited soil disturbance under these systems than CT system. Intensive tillage in CT systems increases decomposition and mineralization of soil organic matter leading to carbon loss, while the practice of CA promotes organic carbon stabilization (Juo and Lal, 1979; Agboola, 1981; Umar et al., 2011).

Cropping system can affect soil C by increased biomass production and carbon inputs from the different crops in the system, among others (Palm et al., 2014). In this study, cropping systems had no significant effect on SOC, although soybean–maize annual rotation and soybean/maize intercropping had 20 and 29% higher SOC content, respectively, compared with sole maize cropping. Similarly, TN content was higher with soybean–maize and intercropping compared with sole cropping. The higher total N in soybean–maize rotation and intercropping systems than in the sole cropping can be attributed to the retention of soybean residues containing high N concentration. Mineral nitrogen levels were lower with CT sole cropping than minimal or NT with soybean–maize rotation or intercropping. The higher levels of mineral N were as a result of NT and the soybean in the rotation and intercropping treatments fixing atmospheric nitrogen.

In the farmers’ fields (baby trials), the difference in either SOC or total N content between no-till and CT plots were not significant after 4 years. However, the data showed an increasing trend in both SOC and total N contents with NT soybean–maize rotation and intercropping compared with CT plots. Statistical insignificance in SOC and TSN contents in response to tillage and cropping systems was likely due to the low crop residue production coupled with removal of all soybean plants from the field for threshing. In farmers’ fields, since crop residues were left in the field after each harvest and not protected, a large portion would be consumed by free roaming livestock, termites and other soil arthropods during the long dry season. Consequently the amount of residue being incorporated at the beginning of the rainy season may have been insufficient to produce a measurable beneficial effect.

### Profitability of CA Practices for Farmers

There must be positive net economic or other benefits to induce a farmer to use a technology. Farmers are most likely to adopt CA when it reduces production costs and/or increases yields, and also when it reduces or at least does not increase risk. In all cropping seasons and cropping systems, total variable costs of production of either maize or soybean were 20–29% lower with NT compared with CT due to lower cost of herbicide for land preparation and weed control and higher cost of plowing. Even though cost of production was lower with NT, average gross benefits for NT sole maize production were lower in the first 3 years when compared to CT. In the fourth year, gross benefit for NT maize production was higher than CT maize production. However, the benefit-to-cost ratios show that both CT and NT continuous maize production were not profitable. For the soybean–maize rotation cropping system, the benefit-to-cost ratios show that in 2010 and 2012, NT sole soybean was more profitable than CT sole soybean. For maize following soybean in the rotation, NT was less profitable in 2011 but more profitable in 2013 when compared to CT. The higher gross margins and profitability of sole soybean when compared to sole maize was because of ready market and higher price offered by the NGO that introduced the crop to the farmers. In 2011 and 2013, CT and NT maize following soybean in the rotation were 2–3 times more profitable than continuous maize cropping under either conventional or NT production. This was due to higher grain yield as a result of the legume benefit to the succeeding maize crop. Hence, it appears that NT is profitable over time for continuous maize and maize following soybean in the rotation.

## Summary and Conclusion

The impact of NT, CT and cropping systems on soil quality and crop productivity was measured during four seasons under researcher and farmer managed conditions. In the researcher managed mother trial, the results showed that the CA practices of NT, residue retention and crop rotation/intercropping can maintain higher soil quality compared to conventional practices. The higher SOC and TNC contents under NT suggest that switching from conventional moldboard plowing to NT can maintain or improve SOC. No significant increase in soil quality indicators was detected in farmers’ fields mainly due to insufficient biomass production, difficulty in residue retention and the practice of removing all soybean plants for threshing outside the fields. Our results showed that in the researcher managed mother trial, tillage and cropping systems did not have a significant impact on maize or soybean yields in the first three seasons. Crop rotation had the greatest impact on maize yields in 2013 with CT rotations increasing maize yields compared to NT maize. In the farmers’ managed trials, CT crop rotation increased maize and soybean yield compared with CA practice of NT and crop rotation. The results suggests that rotation should be an integral part of farmers’ cropping practices and thus for the full benefits of CA to be achieved farmers need to move from continuous mono-cropping to rotations that include legumes. Although our results show a yield advantage of CT cropping systems over NT cropping systems, partial budget analysis showed that the cost of producing maize or soybean is cheaper with NT systems and earns more than double returns to labor than with CT practice. Benefit-to-cost ratios also show that continuous NT soybean and NT soybean–maize rotations are more profitable than CT systems. We conclude that with time, implementation of CA practices involving crop rotation and intercropping of maize and soybean and NT along with crop residue retention presents a win-win scenario due to improved crop yield, increased economic return, and trends of increasing soil fertility. Thus farmers are more likely to adopt NT cropping systems than CT cropping systems. Indeed adoption studies carried out in 2014 showed that 60% of farmers who participated in the on-farm trials adopted the NT and soybean–maize rotation with crop residue retention. For non-participating farmers, the adoption rate was 50%. The average acreage under no-till adoption was found to be 3 acres among all the adopters (Dalton et al., 2014).

## Author Contributions

JN, conceived, designed and conducted the experiment and wrote the manuscript. GM, conducted the experiment and contributed to writing manuscript. IY, conducted economic analysis and contributed to writing manuscript. PP, conceived and designed the experiment, and edited the manuscript.

## Conflict of Interest Statement

The authors declare that the research was conducted in the absence of any commercial or financial relationships that could be construed as a potential conflict of interest.
