# Supplementary material for: Conservation Agriculture Improves Soil Quality, Crop Yield, and Incomes of Smallholder Farmers in North Western Ghana
Source: Front Plant Sci. 2017 Jun 21;8:996. doi: 10.3389/fpls.2017.00996 (PMC5478712; doi:10.3389/fpls.2017.00996)
Supplement: Supplementary file 1 [file Data_Sheet_1.DOCX]

**Two Way Analysis of Variance** Sunday, May 29, 2016, 7:44:04 AM

**Data source:** Bulk density data in 2014 ANALYSIS.JNB

Balanced Design

Dependent Variable: BD

**Normality Test (Shapiro-Wilk)** Passed (P = 0.757)

**Equal Variance Test:** Passed (P = 0.914)

**Source of Variation DF SS MS F P**

TS 2 0.199 0.0997 3.570 0.049

CS 2 0.0434 0.0217 0.777 0.474

TS x CS 4 0.0137 0.00342 0.122 0.973

Residual 18 0.503 0.0279

Total 26 0.759 0.0292

The difference in the mean values among the different levels of TS is greater than would be expected by chance after allowing for effects of differences in CS. There is a statistically significant difference (P = 0.049). To isolate which group(s) differ from the others use a multiple comparison procedure.

The difference in the mean values among the different levels of CS is not great enough to exclude the possibility that the difference is just due to random sampling variability after allowing for the effects of differences in TS. There is not a statistically significant difference (P = 0.474).

The effect of different levels of TS does not depend on what level of CS is present. There is not a statistically significant interaction between TS and CS. (P = 0.973)

Power of performed test with alpha = 0.0500: for TS : 0.438

Power of performed test with alpha = 0.0500: for CS : 0.0500

Power of performed test with alpha = 0.0500: for TS x CS : 0.0500

Least square means for TS :

**Group Mean**

NT 1.697

AT 1.593

CT 1.487

Std Err of LS Mean = 0.0557

Least square means for CS :

**Group Mean**

MZ-MZ 1.591

SB-MZ 1.642

SB/MZ 1.544

Std Err of LS Mean = 0.0557

Least square means for TS x CS :

**Group Mean**

NT x MZ-MZ 1.659

NT x SB-MZ 1.757

NT x SB/MZ 1.677

AT x MZ-MZ 1.618

AT x SB-MZ 1.650

AT x SB/MZ 1.510

CT x MZ-MZ 1.496

CT x SB-MZ 1.520

CT x SB/MZ 1.445

Std Err of LS Mean = 0.0965

**Three Way Analysis of Variance** Thursday, May 26, 2016, 11:29:33 PM

**Data source:** Soil Data 10 in 2014 ANALYSIS.JNB

Balanced Design

Dependent Variable: SOC

**Normality Test (Shapiro-Wilk)** Passed (P = 0.122)

**Equal Variance Test:** Passed (P = 0.126)

**Source of Variation DF SS MS F P**

TS 2 0.138 0.0689 5.644 0.007

CS 2 0.0429 0.0215 1.759 0.187

LAYER 1 0.0122 0.0122 0.997 0.325

TS x CS 4 0.0800 0.0200 1.640 0.186

TS x LAYER 2 0.00159 0.000793 0.0650 0.937

CS x LAYER 2 0.00680 0.00340 0.279 0.758

TS x CS x LAYER 4 0.0492 0.0123 1.009 0.416

Residual 36 0.439 0.0122

Total 53 0.770 0.0145

The difference in the mean values among the different levels of TS are greater than would be expected by chance after allowing for the effects of differences in CS and LAYER. There is a statistically significant difference (P = 0.007). To isolate which group(s) differ from the others use a multiple comparison procedure.

The difference in the mean values among the different levels of CS are not great enough to exclude the possibility that the difference is just due to random sampling variability after allowing for the effects of differences in TS and LAYER. There is not a statistically significant difference (P = 0.187).

The difference in the mean values among the different levels of LAYER are not great enough to exclude the possibility that the difference is just due to random sampling variability after allowing for the effects of differences in TS and CS. There is not a statistically significant difference (P = 0.325).

The effect of different levels of TS does not depend on what level of CS is present. There is not a statistically significant interaction between TS and CS. (P = 0.186)

The effect of different levels of TS does not depend on what level of LAYER is present. There is not a statistically significant interaction between TS and LAYER. (P = 0.937)

The effect of different levels of CS does not depend on what level of LAYER is present. There is not a statistically significant interaction between CS and LAYER. (P = 0.758)

Power of performed test with alpha = 0.0500: for TS : 0.752

Power of performed test with alpha = 0.0500: for CS : 0.162

Power of performed test with alpha = 0.0500: for LAYER : 0.0500

Power of performed test with alpha = 0.0500: for TS x CS : 0.185

Power of performed test with alpha = 0.0500: for TS x LAYER : 0.0500

Power of performed test with alpha = 0.0500: for CS x LAYER : 0.0500

Least square means for TS :

**Group Mean**

CT 0.417

MT 0.526

NT 0.522

Std Err of LS Mean = 0.0260

Least square means for CS :

**Group Mean**

Mz-Mz 0.455

Sb/Mz 0.524

Sb-Mz 0.487

Std Err of LS Mean = 0.0260

Least square means for LAYER :

**Group Mean**

1.000 0.503

2.000 0.473

Std Err of LS Mean = 0.0213

Least square means for TS x CS :

**Group Mean**

CT x Mz-Mz 0.342

CT x Sb/Mz 0.482

CT x Sb-Mz 0.426

MT x Mz-Mz 0.481

MT x Sb/Mz 0.602

MT x Sb-Mz 0.495

NT x Mz-Mz 0.540

NT x Sb/Mz 0.487

NT x Sb-Mz 0.539

Std Err of LS Mean = 0.0451

**Three Way Analysis of Variance** Thursday, May 26, 2016, 11:32:33 PM

**Data source:** Soil Data 10 in 2014ANALYSIS.JNB

Balanced Design

Dependent Variable: TSN

**Normality Test (Shapiro-Wilk)** Passed (P = 0.407)

**Equal Variance Test:** Passed (P = 0.214)

**Source of Variation DF SS MS F P**

TS 2 0.00620 0.00310 43.574 <0.001

CS 2 0.000671 0.000336 4.720 0.015

LAYER 1 0.000383 0.000383 5.385 0.056

TS x CS 4 0.000151 0.0000377 0.531 0.714

TS x LAYER 2 0.0000664 0.0000332 0.467 0.631

CS x LAYER 2 0.0000195 0.00000973 0.137 0.873

TS x CS x LAYER 4 0.000204 0.0000510 0.718 0.585

Residual 36 0.00256 0.0000711

Total 53 0.0103 0.000193

The difference in the mean values among the different levels of TS are greater than would be expected by chance after allowing for the effects of differences in CS and LAYER. There is a statistically significant difference (P = <0.001). To isolate which group(s) differ from the others use a multiple comparison procedure.

The difference in the mean values among the different levels of CS are greater than would be expected by chance after allowing for the effects of differences in TS and LAYER. There is a statistically significant difference (P = 0.015). To isolate which group(s) differ from the others use a multiple comparison procedure.

The difference in the mean values among the different levels of LAYER are greater than would be expected by chance after allowing for the effects of differences in TS and CS. There is a statistically significant difference (P = 0.026). To isolate which group(s) differ from the others use a multiple comparison procedure.

The effect of different levels of TS does not depend on what level of CS is present. There is not a statistically significant interaction between TS and CS. (P = 0.714)

The effect of different levels of TS does not depend on what level of LAYER is present. There is not a statistically significant interaction between TS and LAYER. (P = 0.631)

The effect of different levels of CS does not depend on what level of LAYER is present. There is not a statistically significant interaction between CS and LAYER. (P = 0.873)

All Pairwise Multiple Comparison Procedures (Holm-Sidak method):

Overall significance level = 0.05

Power of performed test with alpha = 0.0500: for TS : 1.000

Power of performed test with alpha = 0.0500: for CS : 0.643

Power of performed test with alpha = 0.0500: for LAYER : 0.523

Power of performed test with alpha = 0.0500: for TS x CS : 0.0500

Power of performed test with alpha = 0.0500: for TS x LAYER : 0.0500

Power of performed test with alpha = 0.0500: for CS x LAYER : 0.0500

Least square means for TS :

**Group Mean**

CT 0.0403

MT 0.0600

NT 0.0652

Std Err of LS Mean = 0.00199

Least square means for CS :

**Group Mean**

Mz-Mz 0.0502

Sb/Mz 0.0575

Sb-Mz 0.0578

Std Err of LS Mean = 0.00199

Least square means for LAYER :

**Group Mean**

1.000 0.0578

2.000 0.0525

Std Err of LS Mean = 0.00162

Least square means for TS x CS :

**Group Mean**

CT x Mz-Mz 0.0372

CT x Sb/Mz 0.0428

CT x Sb-Mz 0.0410

MT x Mz-Mz 0.0543

MT x Sb/Mz 0.0640

MT x Sb-Mz 0.0616

NT x Mz-Mz 0.0590

NT x Sb/Mz 0.0658

NT x Sb-Mz 0.0708

Std Err of LS Mean = 0.00344

**Three Way Analysis of Variance** Friday, May 27, 2016, 12:21:56 AM

**Data source:** Soil Data 10 in 2014 ANALYSIS.JNB

Balanced Design

Dependent Variable: Nmin

**Normality Test (Shapiro-Wilk)** Passed (P = 0.711)

**Equal Variance Test:** Passed (P = 0.699)

**Source of Variation DF SS MS F P**

TS 2 3946.141 1973.071 35.643 <0.001

CS 2 244.336 122.168 2.207 0.125

LAYER 1 98.443 98.443 1.778 0.191

TS x CS 4 951.495 237.874 4.297 0.006

TS x LAYER 2 360.969 180.484 3.260 0.050

CS x LAYER 2 174.502 87.251 1.576 0.221

TS x CS x LAYER 4 65.343 16.336 0.295 0.879

Residual 36 1992.807 55.356

Total 53 7834.036 147.812

The main effects for TS cannot be properly interpreted since the size of the factor's effect depends upon the level of another factor.

The main effects for CS cannot be properly interpreted since the size of the factor's effect depends upon the level of another factor.

The main effects for LAYER cannot be properly interpreted since the size of the factor's effect depends upon the level of another factor.

The effect of different levels of TS depends on what level of CS is present. There is a statistically significant interaction between TS and CS. (P = 0.006)

The effect of different levels of TS depends on what level of LAYER is present. There is a statistically significant interaction between TS and LAYER. (P = 0.050)

The effect of different levels of CS does not depend on what level of LAYER is present. There is not a statistically significant interaction between CS and LAYER. (P = 0.221)

All Pairwise Multiple Comparison Procedures (Holm-Sidak method):

Overall significance level = 0.05

Power of performed test with alpha = 0.0500: for TS : 1.000

Power of performed test with alpha = 0.0500: for CS : 0.237

Power of performed test with alpha = 0.0500: for LAYER : 0.129

Power of performed test with alpha = 0.0500: for TS x CS : 0.790

Power of performed test with alpha = 0.0500: for TS x LAYER : 0.419

Power of performed test with alpha = 0.0500: for CS x LAYER : 0.133

Least square means for TS :

**Group Mean**

CT 23.626

MT 35.486

NT 44.501

Std Err of LS Mean = 1.754

Least square means for CS :

**Group Mean**

Mz-Mz 31.942

Sb/Mz 34.517

Sb-Mz 37.152

Std Err of LS Mean = 1.754

Least square means for LAYER :

**Group Mean**

1.000 33.187

2.000 35.888

Std Err of LS Mean = 1.432

Least square means for TS x CS :

**Group Mean**

CT x Mz-Mz 20.280

CT x Sb/Mz 24.015

CT x Sb-Mz 26.582

MT x Mz-Mz 40.483

MT x Sb/Mz 32.171

MT x Sb-Mz 33.804

NT x Mz-Mz 35.064

NT x Sb/Mz 47.367

NT x Sb-Mz 51.071

Std Err of LS Mean = 3.037

**Two Way Analysis of Variance** Sunday, April 14, 2013, 3:28:35 PM

**Data source:** Maize in 2010-ANALYSIS.JNB

Balanced Design

Dependent Variable: RESIDUE

**Normality Test (Shapiro-Wilk)** Passed (P = 0.627)

**Equal Variance Test:** Passed (P = 0.336)

**Source of Variation DF SS MS F P**

TS 2 922133.531 461066.765 1.126 0.356

CS 1 17422.219 17422.219 0.0425 0.840

TS x CS 2 331911.088 165955.544 0.405 0.676

Residual 12 4915733.310 409644.443

Total 17 6187200.148 363952.950

The difference in the mean values among the different levels of TS is not great enough to exclude the possibility that the difference is just due to random sampling variability after allowing for the effects of differences in CS. There is not a statistically significant difference (P = 0.356).

The difference in the mean values among the different levels of CS is not great enough to exclude the possibility that the difference is just due to random sampling variability after allowing for the effects of differences in TS. There is not a statistically significant difference (P = 0.840).

The effect of different levels of TS does not depend on what level of CS is present. There is not a statistically significant interaction between TS and CS. (P = 0.676)

Power of performed test with alpha = 0.0500: for TS : 0.0641

Power of performed test with alpha = 0.0500: for CS : 0.0500

Power of performed test with alpha = 0.0500: for TS x CS : 0.0500

Least square means for TS :

**Group Mean**

CT 2106.667

MT 2300.000

NT 1753.333

Std Err of LS Mean = 261.293

Least square means for CS :

**Group Mean**

CM 2084.444

S/M 2022.222

Std Err of LS Mean = 213.345

Least square means for TS x CS :

**Group Mean**

CT x CM 2013.333

CT x S/M 2200.000

MT x CM 2266.667

MT x S/M 2333.333

NT x CM 1973.333

NT x S/M 1533.333

Std Err of LS Mean = 369.524

**Two Way Analysis of Variance** Friday, September 21, 2012, 4:34:04 PM

**Data source:** Nyoli-mother in 2011-ANALYSIS.JNB

Balanced Design

Dependent Variable: Residues

**Normality Test (Shapiro-Wilk)** Passed (P = 0.100)

**Equal Variance Test:** Passed (P = 0.969)

**Source of Variation DF SS MS F P**

TS 2 243762.906 121881.453 0.464 0.636

CS 2 684474.034 342237.017 1.303 0.296

TS x CS 4 815259.323 203814.831 0.776 0.555

Residual 18 4726666.489 262592.583

Total 26 6470162.752 248852.414

The difference in the mean values among the different levels of TS is not great enough to exclude the possibility that the difference is just due to random sampling variability after allowing for the effects of differences in CS. There is not a statistically significant difference (P = 0.636).

The difference in the mean values among the different levels of CS is not great enough to exclude the possibility that the difference is just due to random sampling variability after allowing for the effects of differences in TS. There is not a statistically significant difference (P = 0.296).

The effect of different levels of TS does not depend on what level of CS is present. There is not a statistically significant interaction between TS and CS. (P = 0.555)

Power of performed test with alpha = 0.0500: for TS : 0.0500

Power of performed test with alpha = 0.0500: for CS : 0.0886

Power of performed test with alpha = 0.0500: for TS x CS : 0.0500

Least square means for TS :

**Group Mean**

CT 2004.444

AT 2042.222

NT 1824.444

Std Err of LS Mean = 170.813

Least square means for CS :

**Group Mean**

CM 2046.667

Mz/Sb 2091.111

Mz-Sb 1733.333

Std Err of LS Mean = 170.813

Least square means for TS x CS :

**Group Mean**

CT x CM 2386.667

CT x Mz/Sb 2066.667

CT x Mz-Sb 1560.000

AT x CM 1860.000

AT x Mz/Sb 2213.333

AT x Mz-Sb 2053.333

NT x CM 1893.333

NT x Mz/Sb 1993.333

NT x Mz-Sb 1586.667

Std Err of LS Mean = 295.856

**Two Way Analysis of Variance** Sunday, April 14, 2013, 12:35:10 PM

**Data source:** Maize data in 2012 Mother Trial ANALYSIS.JNB

Balanced Design

Dependent Variable: MZ STOV

**Normality Test (Shapiro-Wilk)** Passed (P = 0.350)

**Equal Variance Test:** Passed (P = 0.612)

**Source of Variation DF SS MS F P**

TS 2 1987600.000 993800.000 2.122 0.163

CS 1 4032800.000 4032800.000 8.610 0.013

TS x CS 2 293200.000 146600.000 0.313 0.737

Residual 12 5620800.000 468400.000

Total 17 11934400.000 702023.529

The difference in the mean values among the different levels of TS is not great enough to exclude the possibility that the difference is just due to random sampling variability after allowing for the effects of differences in CS. There is not a statistically significant difference (P = 0.163).

The difference in the mean values among the different levels of CS is greater than would be expected by chance after allowing for effects of differences in TS. There is a statistically significant difference (P = 0.013). To isolate which group(s) differ from the others use a multiple comparison procedure.

The effect of different levels of TS does not depend on what level of CS is present. There is not a statistically significant interaction between TS and CS. (P = 0.737)

Power of performed test with alpha = 0.0500: for TS : 0.196

Power of performed test with alpha = 0.0500: for CS : 0.723

Power of performed test with alpha = 0.0500: for TS x CS : 0.0500

Least square means for TS :

**Group Mean**

CT 3070.000

MT 2270.000

NT 2540.000

Std Err of LS Mean = 279.404

Least square means for CS :

**Group Mean**

CM 2153.333

M/S 3100.000

Std Err of LS Mean = 228.133

Least square means for TS x CS :

**Group Mean**

CT x CM 2620.000

CT x M/S 3520.000

MT x CM 1940.000

MT x M/S 2600.000

NT x CM 1900.000

NT x M/S 3180.000

Std Err of LS Mean = 395.137

**Two Way Analysis of Variance** Tuesday, June 14, 2016, 1:03:13 PM

**Data source:** Nyoli-Mother in 2013-ANALYSIS.JNB

Balanced Design

Dependent Variable: Residue

**Normality Test (Shapiro-Wilk)** Passed (P = 0.585)

**Equal Variance Test:** Passed (P = 0.579)

**Source of Variation DF SS MS F P**

TS 2 4162005.740 2081002.870 26.478 <0.001

CS 2 1584125.123 792062.561 10.078 0.001

TS x CS 4 618100.745 154525.186 1.966 0.143

Residual 18 1414677.204 78593.178

Total 26 7778908.811 299188.800

The difference in the mean values among the different levels of TS is greater than would be expected by chance after allowing for effects of differences in CS. There is a statistically significant difference (P = <0.001). To isolate which group(s) differ from the others use a multiple comparison procedure.

The difference in the mean values among the different levels of CS is greater than would be expected by chance after allowing for effects of differences in TS. There is a statistically significant difference (P = 0.001). To isolate which group(s) differ from the others use a multiple comparison procedure.

The effect of different levels of TS does not depend on what level of CS is present. There is not a statistically significant interaction between TS and CS. (P = 0.143)

Power of performed test with alpha = 0.0500: for TS : 1.000

Power of performed test with alpha = 0.0500: for CS : 0.956

Power of performed test with alpha = 0.0500: for TS x CS : 0.241

Least square means for TS :

**Group Mean**

CT 2875.111

MT 2232.091

NT 1934.278

Std Err of LS Mean = 93.448

Least square means for CS :

**Group Mean**

CM 2155.500

Mz/Sb 2197.110

Mz-Sb 2688.869

Std Err of LS Mean = 93.448

Least square means for TS x CS :

**Group Mean**

CT x CM 2436.667

CT x Mz/Sb 2720.000

CT x Mz-Sb 3468.667

MT x CM 2121.500

MT x Mz/Sb 2165.163

MT x Mz-Sb 2409.608

NT x CM 1908.333

NT x Mz/Sb 1706.167

NT x Mz-Sb 2188.333

Std Err of LS Mean = 161.857

**Two Way Analysis of Variance** Tuesday, May 24, 2016, 8:09:07 PM

**Data source:** Maize in 2010-ANALYSIS.JNB

Balanced Design

Dependent Variable: GYIELD

**Normality Test (Shapiro-Wilk)** Passed (P = 0.394)

**Equal Variance Test:** Passed (P = 0.058)

**Source of Variation DF SS MS F P**

TS 2 816273.818 408136.909 1.437 0.276

CS 1 22597.800 22597.800 0.0796 0.783

TS x CS 2 990792.353 495396.177 1.744 0.216

Residual 12 3407865.200 283988.767

Total 17 5237529.171 308089.951

The difference in the mean values among the different levels of TS is not great enough to exclude the possibility that the difference is just due to random sampling variability after allowing for the effects of differences in CS. There is not a statistically significant difference (P = 0.276).

The difference in the mean values among the different levels of CS is not great enough to exclude the possibility that the difference is just due to random sampling variability after allowing for the effects of differences in TS. There is not a statistically significant difference (P = 0.783).

The effect of different levels of TS does not depend on what level of CS is present. There is not a statistically significant interaction between TS and CS. (P = 0.216)

Power of performed test with alpha = 0.0500: for TS : 0.103

Power of performed test with alpha = 0.0500: for CS : 0.0500

Power of performed test with alpha = 0.0500: for TS x CS : 0.143

Least square means for TS :

**Group Mean**

CT 1903.704

MT 1687.037

NT 1384.444

Std Err of LS Mean = 217.558

Least square means for CS :

**Group Mean**

CM 1622.963

S/M 1693.827

Std Err of LS Mean = 177.635

Least square means for TS x CS :

**Group Mean**

CT x CM 1955.556

CT x S/M 1851.852

MT x CM 1885.185

MT x S/M 1488.889

NT x CM 1028.148

NT x S/M 1740.741

Std Err of LS Mean = 307.673

**Two Way Analysis of Variance** Tuesday, May 24, 2016, 7:44:43 PM

**Data source:** Soybean in 2010-ANALYSIS.JNB

Balanced Design

Dependent Variable: SB-GY2

**Normality Test (Shapiro-Wilk)** Passed (P = 0.155)

**Equal Variance Test:** Passed (P = 0.829)

**Source of Variation DF SS MS F P**

TS 2 101124.829 50562.414 0.544 0.594

CS 1 545432.099 545432.099 5.865 0.032

TS x CS 2 34732.510 17366.255 0.187 0.832

Residual 12 1116049.383 93004.115

Total 17 1797338.820 105725.813

The difference in the mean values among the different levels of TS is not great enough to exclude the possibility that the difference is just due to random sampling variability after allowing for the effects of differences in CS. There is not a statistically significant difference (P = 0.594).

The difference in the mean values among the different levels of CS is greater than would be expected by chance after allowing for effects of differences in TS. There is a statistically significant difference (P = 0.032). To isolate which group(s) differ from the others use a multiple comparison procedure.

The effect of different levels of TS does not depend on what level of CS is present. There is not a statistically significant interaction between TS and CS. (P = 0.832)

Power of performed test with alpha = 0.0500: for TS : 0.0500

Power of performed test with alpha = 0.0500: for CS : 0.522

Power of performed test with alpha = 0.0500: for TS x CS : 0.0500

Least square means for TS :

**Group Mean**

CT 1222.222

MT 1288.889

NT 1107.407

Std Err of LS Mean = 124.502

Least square means for CS :

**Group Mean**

SB-MZ 1380.247

S/M 1032.099

Std Err of LS Mean = 101.655

Least square means for TS x CS :

**Group Mean**

CT x SB-MZ 1392.593

CT x S/M 1051.852

MT x SB-MZ 1518.519

MT x S/M 1059.259

NT x SB-MZ 1229.630

NT x S/M 985.185

Std Err of LS Mean = 176.072

**Two Way Analysis of Variance** Sunday, April 14, 2013, 6:04:34 PM

**Data source:** Maize in 2012 ANALYSIS

Balanced Design

Dependent Variable: MZ-GY

**Normality Test (Shapiro-Wilk)** Passed (P = 0.478)

**Equal Variance Test:** Passed (P = 0.683)

**Source of Variation DF SS MS F P**

TS 2 1775916.444 887958.222 4.773 0.030

CS 1 27222.222 27222.222 0.146 0.709

TS x CS 2 75841.778 37920.889 0.204 0.818

Residual 12 2232426.667 186035.556

Total 17 4111407.111 241847.477

The difference in the mean values among the different levels of TS is greater than would be expected by chance after allowing for effects of differences in CS. There is a statistically significant difference (P = 0.030). To isolate which group(s) differ from the others use a multiple comparison procedure.

The difference in the mean values among the different levels of CS is not great enough to exclude the possibility that the difference is just due to random sampling variability after allowing for the effects of differences in TS. There is not a statistically significant difference (P = 0.709).

The effect of different levels of TS does not depend on what level of CS is present. There is not a statistically significant interaction between TS and CS. (P = 0.818)

Power of performed test with alpha = 0.0500: for TS : 0.569

Power of performed test with alpha = 0.0500: for CS : 0.0500

Power of performed test with alpha = 0.0500: for TS x CS : 0.0500

Least square means for TS :

**Group Mean**

CT 1698.667

MT 1004.667

NT 1064.000

Std Err of LS Mean = 176.085

Least square means for CS :

**Group Mean**

CM 1294.667

M/S 1216.889

Std Err of LS Mean = 143.773

Least square means for TS x CS :

**Group Mean**

CT x CM 1693.333

CT x M/S 1704.000

MT x CM 996.000

MT x M/S 1013.333

NT x CM 1194.667

NT x M/S 933.333

Std Err of LS Mean = 249.022

**Two Way Analysis of Variance** Sunday, October 13, 2013, 4:59:08 PM

**Data source:** Soybean data in 2012 ANALYSIS

Balanced Design

Dependent Variable: SB-GY

**Normality Test (Shapiro-Wilk)** Passed (P = 0.302)

**Equal Variance Test:** Passed (P = 0.179)

**Source of Variation DF SS MS F P**

TS 2 457012.000 228506.000 2.148 0.159

CS 1 24642.000 24642.000 0.232 0.639

TS x CS 2 85044.000 42522.000 0.400 0.679

Residual 12 1276704.000 106392.000

Total 17 1843402.000 108435.412

The difference in the mean values among the different levels of TS is not great enough to exclude the possibility that the difference is just due to random sampling variability after allowing for the effects of differences in CS. There is not a statistically significant difference (P = 0.159).

The difference in the mean values among the different levels of CS is not great enough to exclude the possibility that the difference is just due to random sampling variability after allowing for the effects of differences in TS. There is not a statistically significant difference (P = 0.639).

The effect of different levels of TS does not depend on what level of CS is present. There is not a statistically significant interaction between TS and CS. (P = 0.679)

Power of performed test with alpha = 0.0500: for TS : 0.200

Power of performed test with alpha = 0.0500: for CS : 0.0500

Power of performed test with alpha = 0.0500: for TS x CS : 0.0500

Least square means for TS :

**Group Mean**

CT 1402.000

MT 1350.000

NT 1041.000

Std Err of LS Mean = 133.162

Least square means for CS :

**Group Mean**

M/S 1301.333

S-M 1227.333

Std Err of LS Mean = 108.726

Least square means for TS x CS :

**Group Mean**

CT x M/S 1396.000

CT x S-M 1408.000

MT x M/S 1484.000

MT x S-M 1216.000

NT x M/S 1024.000

NT x S-M 1058.000

Std Err of LS Mean = 188.319

**Two Way Analysis of Variance** Tuesday, May 06, 2014, 9:58:50 PM

**Data source:** Nyoli-mother in 2011-ANALYSIS

Balanced Design

Dependent Variable: GY

**Normality Test (Shapiro-Wilk)** Passed (P = 0.808)

**Equal Variance Test:** Passed (P = 0.612)

**Source of Variation DF SS MS F P**

TS 2 545807.407 272903.704 1.567 0.236

CS 2 350742.519 175371.259 1.007 0.385

TS x CS 4 123405.037 30851.259 0.177 0.947

Residual 18 3135093.333 174171.852

Total 26 4155048.296 159809.550

The difference in the mean values among the different levels of TS is not great enough to exclude the possibility that the difference is just due to random sampling variability after allowing for the effects of differences in CS. There is not a statistically significant difference (P = 0.236).

The difference in the mean values among the different levels of CS is not great enough to exclude the possibility that the difference is just due to random sampling variability after allowing for the effects of differences in TS. There is not a statistically significant difference (P = 0.385).

The effect of different levels of TS does not depend on what level of CS is present. There is not a statistically significant interaction between TS and CS. (P = 0.947)

Power of performed test with alpha = 0.0500: for TS : 0.125

Power of performed test with alpha = 0.0500: for CS : 0.0500

Power of performed test with alpha = 0.0500: for TS x CS : 0.0500

Least square means for TS :

**Group Mean**

CT 1581.333

AT 1234.667

NT 1379.111

Std Err of LS Mean = 139.113

Least square means for CS :

**Group Mean**

CM 1317.333

Mz/Sb 1318.222

Mz-Sb 1559.556

Std Err of LS Mean = 139.113

Least square means for TS x CS :

**Group Mean**

CT x CM 1557.333

CT x Mz/Sb 1386.667

CT x Mz-Sb 1800.000

AT x CM 1081.333

AT x Mz/Sb 1272.000

AT x Mz-Sb 1350.667

NT x CM 1313.333

NT x Mz/Sb 1296.000

NT x Mz-Sb 1528.000

Std Err of LS Mean = 240.951

**Two Way Analysis of Variance** Wednesday, May 25, 2016, 11:30:57 AM

**Data source:** Nyoli-Mother in 2013-ANALYSIS.JNB

Balanced Design

Dependent Variable: MZ-GY3

**Normality Test (Shapiro-Wilk)** Passed (P = 0.662)

**Equal Variance Test:** Passed (P = 0.807)

**Source of Variation DF SS MS F P**

TS 2 8639618.107 4319809.053 47.286 <0.001

CS 2 3967035.391 1983517.695 21.712 <0.001

TS x CS 4 1412826.337 353206.584 3.866 0.019

Residual 18 1644385.185 91354.733

Total 26 15663865.021 602456.347

Main effects cannot be properly interpreted if significant interaction is determined. This is because the size of a factor's effect depends upon the level of the other factor.

The effect of different levels of TS depends on what level of CS is present. There is a statistically significant interaction between TS and CS. (P = 0.019)

Power of performed test with alpha = 0.0500: for TS : 1.000

Power of performed test with alpha = 0.0500: for CS : 1.000

Power of performed test with alpha = 0.0500: for TS x CS : 0.655

Least square means for TS :

**Group Mean**

CT 2625.185

MT 1537.778

NT 1337.778

Std Err of LS Mean = 100.750

Least square means for CS :

**Group Mean**

CM 1959.259

Mz/Sb 1314.074

Mz-Sb 2227.407

Std Err of LS Mean = 100.750

Least square means for TS x CS :

**Group Mean**

CT x CM 2891.111

CT x Mz/Sb 1662.222

CT x Mz-Sb 3322.222

MT x CM 1564.444

MT x Mz/Sb 1293.333

MT x Mz-Sb 1755.556

NT x CM 1422.222

NT x Mz/Sb 986.667

NT x Mz-Sb 1604.444

Std Err of LS Mean = 174.504

*************************************BABY TRIALS ANOVA********************************

**Two Way Analysis of Variance** Tuesday, June 14, 2016, 10:33:20 PM

**Data source:** Nyoli Babies BD in 2014-ANALYSIS.JNB

Balanced Design

Dependent Variable: BD

**Normality Test (Shapiro-Wilk)** Passed (P = 0.589)

**Equal Variance Test:** Passed (P = 0.756)

**Source of Variation DF SS MS F P**

TS 1 0.0897 0.0897 13.291 0.003

CSYS 1 0.00854 0.00854 1.266 0.282

TS x CSYS 1 0.00140 0.00140 0.207 0.657

Residual 12 0.0810 0.00675

Total 15 0.181 0.0120

The difference in the mean values among the different levels of TS is greater than would be expected by chance after allowing for effects of differences in CSYS. There is a statistically significant difference (P = 0.003). To isolate which group(s) differ from the others use a multiple comparison procedure.

The difference in the mean values among the different levels of CSYS is not great enough to exclude the possibility that the difference is just due to random sampling variability after allowing for the effects of differences in TS. There is not a statistically significant difference (P = 0.282).

The effect of different levels of TS does not depend on what level of CSYS is present. There is not a statistically significant interaction between TS and CSYS. (P = 0.657)

Power of performed test with alpha = 0.0500: for TS : 0.909

Power of performed test with alpha = 0.0500: for CSYS : 0.0730

Power of performed test with alpha = 0.0500: for TS x CSYS : 0.0500

Least square means for TS :

**Group Mean**

NT 1.677

CT 1.527

Std Err of LS Mean = 0.0290

Least square means for CSYS :

**Group Mean**

Mz-Mz 1.625

Sb-Mz 1.579

Std Err of LS Mean = 0.0290

Least square means for TS x CSYS :

**Group Mean**

NT x Mz-Mz 1.691

NT x Sb-Mz 1.663

CT x Mz-Mz 1.560

CT x Sb-Mz 1.495

Std Err of LS Mean = 0.0411

**Two Way Analysis of Variance** Thursday, June 16, 2016, 9:55:18 PM

**Data source:** Soil Data-Babies in 2014 ANALYSIS.JNB

Balanced Design

Dependent Variable: SOC

**Normality Test (Shapiro-Wilk)** Passed (P = 0.374)

**Equal Variance Test:** Passed (P = 0.732)

**Source of Variation DF SS MS F P**

TS 1 0.000705 0.000705 0.135 0.718

CS 1 0.000439 0.000439 0.0842 0.775

TS x CS 1 0.00279 0.00279 0.535 0.475

Residual 16 0.0835 0.00522

Total 19 0.0875 0.00460

The difference in the mean values among the different levels of TS is not great enough to exclude the possibility that the difference is just due to random sampling variability after allowing for the effects of differences in CS. There is not a statistically significant difference (P = 0.718).

The difference in the mean values among the different levels of CS is not great enough to exclude the possibility that the difference is just due to random sampling variability after allowing for the effects of differences in TS. There is not a statistically significant difference (P = 0.775).

The effect of different levels of TS does not depend on what level of CS is present. There is not a statistically significant interaction between TS and CS. (P = 0.475)

Power of performed test with alpha = 0.0500: for TS : 0.0500

Power of performed test with alpha = 0.0500: for CS : 0.0500

Power of performed test with alpha = 0.0500: for TS x CS : 0.0500

Least square means for TS :

**Group Mean**

CT 0.385

NT 0.397

Std Err of LS Mean = 0.0228

Least square means for CS :

**Group Mean**

CM 0.386

SB-MZ 0.396

Std Err of LS Mean = 0.0228

Least square means for TS x CS :

**Group Mean**

CT x CM 0.368

CT x SB-MZ 0.402

NT x CM 0.404

NT x SB-MZ 0.390

Std Err of LS Mean = 0.0323

**Two Way Analysis of Variance** Thursday, June 16, 2016, 9:56:01 PM

**Data source:** Soil Data-Babies in 2014 ANALYSIS.JNB

Balanced Design

Dependent Variable: TSN

**Normality Test (Shapiro-Wilk)** Passed (P = 0.338)

**Equal Variance Test:** Passed (P = 0.904)

**Source of Variation DF SS MS F P**

TS 1 0.00000661 0.00000661 0.175 0.681

CS 1 0.00000451 0.00000451 0.119 0.734

TS x CS 1 0.0000231 0.0000231 0.611 0.446

Residual 16 0.000606 0.0000378

Total 19 0.000640 0.0000337

The difference in the mean values among the different levels of TS is not great enough to exclude the possibility that the difference is just due to random sampling variability after allowing for the effects of differences in CS. There is not a statistically significant difference (P = 0.681).

The difference in the mean values among the different levels of CS is not great enough to exclude the possibility that the difference is just due to random sampling variability after allowing for the effects of differences in TS. There is not a statistically significant difference (P = 0.734).

The effect of different levels of TS does not depend on what level of CS is present. There is not a statistically significant interaction between TS and CS. (P = 0.446)

Power of performed test with alpha = 0.0500: for TS : 0.0500

Power of performed test with alpha = 0.0500: for CS : 0.0500

Power of performed test with alpha = 0.0500: for TS x CS : 0.0500

Least square means for TS :

**Group Mean**

CT 0.0342

NT 0.0353

Std Err of LS Mean = 0.00195

Least square means for CS :

**Group Mean**

CM 0.0343

SB-MZ 0.0352

Std Err of LS Mean = 0.00195

Least square means for TS x CS :

**Group Mean**

CT x CM 0.0326

CT x SB-MZ 0.0357

NT x CM 0.0359

NT x SB-MZ 0.0347

Std Err of LS Mean = 0.00275

**Two Way Analysis of Variance** Thursday, June 16, 2016, 9:56:43 PM

**Data source:** Soil Data-Babies in 2014 ANALYSIS.JNB

Balanced Design

Dependent Variable: NMIN

**Normality Test (Shapiro-Wilk)** Passed (P = 0.214)

**Equal Variance Test:** Passed (P = 0.994)

**Source of Variation DF SS MS F P**

TS 1 0.0551 0.0551 0.00170 0.968

CS 1 33.450 33.450 1.030 0.325

TS x CS 1 0.185 0.185 0.00571 0.941

Residual 16 519.526 32.470

Total 19 553.216 29.117

The difference in the mean values among the different levels of TS is not great enough to exclude the possibility that the difference is just due to random sampling variability after allowing for the effects of differences in CS. There is not a statistically significant difference (P = 0.968).

The difference in the mean values among the different levels of CS is not great enough to exclude the possibility that the difference is just due to random sampling variability after allowing for the effects of differences in TS. There is not a statistically significant difference (P = 0.325).

The effect of different levels of TS does not depend on what level of CS is present. There is not a statistically significant interaction between TS and CS. (P = 0.941)

Power of performed test with alpha = 0.0500: for TS : 0.0500

Power of performed test with alpha = 0.0500: for CS : 0.0508

Power of performed test with alpha = 0.0500: for TS x CS : 0.0500

Least square means for TS :

**Group Mean**

CT 21.802

NT 21.697

Std Err of LS Mean = 1.802

Least square means for CS :

**Group Mean**

CM 20.456

SB-MZ 23.042

Std Err of LS Mean = 1.802

Least square means for TS x CS :

**Group Mean**

CT x CM 20.605

CT x SB-MZ 22.999

NT x CM 20.307

NT x SB-MZ 23.086

Std Err of LS Mean = 2.548

**One Way Analysis of Variance** Sunday, April 14, 2013, 4:24:10 PM

**Data source:** Babies in 2010-ANALYSIS.JNB

Dependent Variable: RESIDUE

**Normality Test (Shapiro-Wilk)** Passed (P = 0.693)

**Equal Variance Test:** Passed (P = 0.318)

**Group Name N Missing Mean Std Dev SEM**

CT 28 0 1788.214 368.632 69.665

NT 28 0 1905.000 481.021 90.904

**Source of Variation DF SS MS F P**

Between Groups 1 190944.643 190944.643 1.040 0.312

Residual 54 9916310.714 183635.384

Total 55 10107255.357

The differences in the mean values among the treatment groups are not great enough to exclude the possibility that the difference is due to random sampling variability; there is not a statistically significant difference (P = 0.312).

Power of performed test with alpha = 0.050: 0.052

The power of the performed test (0.052) is below the desired power of 0.800.

Less than desired power indicates you are less likely to detect a difference when one actually exists. Negative results should be interpreted cautiously.

**Two Way Analysis of Variance** Friday, September 21, 2012, 4:58:45 PM

**Data source:** Nyoli-Babies in 2011-ANALYSIS.JNB

Balanced Design

Dependent Variable: Residues

**Normality Test (Shapiro-Wilk)** Passed (P = 0.497)

**Equal Variance Test:** Passed (P = 0.055)

**Source of Variation DF SS MS F P**

TS 1 3565104.321 3565104.321 7.098 0.009

CS 1 2646704.133 2646704.133 5.269 0.024

TS x CS 1 2142037.500 2142037.500 4.265 0.042

Residual 92 46210750.025 502290.761

Total 95 54564595.979 574364.168

Main effects cannot be properly interpreted if significant interaction is determined. This is because the size of a factor's effect depends upon the level of the other factor.

The effect of different levels of TS depends on what level of CS is present. There is a statistically significant interaction between TS and CS. (P = 0.042)

Power of performed test with alpha = 0.0500: for TS : 0.690

Power of performed test with alpha = 0.0500: for CS : 0.525

Power of performed test with alpha = 0.0500: for TS x CS : 0.417

Least square means for TS :

**Group Mean**

CT 2384.167

NT 1998.750

Std Err of LS Mean = 102.296

Least square means for CS :

**Group Mean**

CM 2025.417

S-M 2357.500

Std Err of LS Mean = 102.296

Least square means for TS x CS :

**Group Mean**

CT x CM 2367.500

CT x S-M 2400.833

NT x CM 1683.333

NT x S-M 2314.167

Std Err of LS Mean = 144.668

**One Way Analysis of Variance** Sunday, April 14, 2013, 1:17:00 PM

**Data source:** Maize in 2012 ANALYSIS.JNB

Dependent Variable: MZ-STOVER

**Normality Test (Shapiro-Wilk)** Passed (P = 0.418)

**Equal Variance Test:** Passed (P = 0.789)

**Group Name N Missing Mean Std Dev SEM**

CT 24 0 2842.083 304.409 62.137

NT 24 0 2221.417 280.807 57.319

**Source of Variation DF SS MS F P**

Between Groups 1 4622722.975 4622722.975 53.904 <0.001

Residual 46 3944889.805 85758.474

Total 47 8567612.780

The differences in the mean values among the treatment groups are greater than would be expected by chance; there is a statistically significant difference (P = <0.001).

Power of performed test with alpha = 0.050: 1.000

All Pairwise Multiple Comparison Procedures (Holm-Sidak method):

Overall significance level = 0.05

Comparisons for factor: **TS**

**Comparison Diff of Means t Unadjusted P Critical Level Significant?**

CT vs. NT 620.667 7.342 <0.001 0.050 Yes

**One Way Analysis of Variance** Tuesday, May 06, 2014, 10:52:40 PM

**Data source:** Babies in 2010-ANALYSIS

Dependent Variable: MZ-GY

**Normality Test (Shapiro-Wilk)** Passed (P = 0.057)

**Equal Variance Test:** Passed (P = 0.171)

**Group Name N Missing Mean Std Dev SEM**

CT 28 0 1294.286 564.712 106.721

NT 28 0 995.714 416.417 78.695

**Source of Variation DF SS MS F P**

Between Groups 1 1248028.571 1248028.571 5.070 0.028

Residual 54 13292171.429 246151.323

Total 55 14540200.000

The differences in the mean values among the treatment groups are greater than would be expected by chance; there is a statistically significant difference (P = 0.028).

Power of performed test with alpha = 0.050: 0.499

All Pairwise Multiple Comparison Procedures (Fisher LSD Method):

Comparisons for factor: **TSYS**

**Comparison Diff of Means LSD(alpha=0.050) P Diff >= LSD**

CT vs. NT 298.571 265.843 0.028 Yes

**One Way Analysis of Variance** Tuesday, May 06, 2014, 11:03:55 PM

**Data source:** Maize in 2012 ANALYSIS

Dependent Variable: MZ-GRAIN

**Normality Test (Shapiro-Wilk)** Passed (P = 0.290)

**Equal Variance Test:** Passed (P = 0.169)

**Group Name N Missing Mean Std Dev SEM**

CT 24 0 1765.000 569.944 116.339

NT 24 0 1108.333 464.821 94.881

**Source of Variation DF SS MS F P**

Between Groups 1 5174533.333 5174533.333 19.133 <0.001

Residual 46 12440577.778 270447.343

Total 47 17615111.111

The differences in the mean values among the treatment groups are greater than would be expected by chance; there is a statistically significant difference (P = <0.001).

Power of performed test with alpha = 0.050: 0.993

All Pairwise Multiple Comparison Procedures (Fisher LSD Method):

Comparisons for factor: **TS**

**Comparison Diff of Means LSD(alpha=0.050) P Diff >= LSD**

CT vs. NT 656.667 302.184 <0.001 Yes

**One Way Analysis of Variance** Tuesday, May 06, 2014, 9:47:12 PM

**Data source:** Babies in 2010-ANALYSIS.JNB

Dependent Variable: SB-GYIELD

**Normality Test (Shapiro-Wilk)** Passed (P = 0.073)

**Equal Variance Test:** Passed (P = 0.168)

**Group Name N Missing Mean Std Dev SEM**

CT 28 0 1234.857 468.553 88.548

NT 28 0 1110.429 368.193 69.582

**Source of Variation DF SS MS F P**

Between Groups 1 216754.571 216754.571 1.221 0.274

Residual 54 9587894.286 177553.598

Total 55 9804648.857

The differences in the mean values among the treatment groups are not great enough to exclude the possibility that the difference is due to random sampling variability; there is not a statistically significant difference (P = 0.274).

Power of performed test with alpha = 0.050: 0.071

The power of the performed test (0.071) is below the desired power of 0.800.

Less than desired power indicates you are less likely to detect a difference when one actually exists. Negative results should be interpreted cautiously.

**One Way Analysis of Variance** Tuesday, May 06, 2014, 7:13:19 PM

**Data source:** Soybean in 2012 ANALYSIS.JNB

Dependent Variable: SB-GY

**Normality Test (Shapiro-Wilk)** Passed (P = 0.742)

**Equal Variance Test:** Passed (P = 0.266)

**Group Name N Missing Mean Std Dev SEM**

CT 24 0 1524.300 406.698 83.017

NT 24 0 1147.500 284.718 58.118

**Source of Variation DF SS MS F P**

Between Groups 1 1703738.880 1703738.880 13.825 <0.001

Residual 46 5668756.960 123233.847

Total 47 7372495.840

The differences in the mean values among the treatment groups are greater than would be expected by chance; there is a statistically significant difference (P = <0.001).

Power of performed test with alpha = 0.050: 0.955

All Pairwise Multiple Comparison Procedures (Holm-Sidak method):

Overall significance level = 0.05

Comparisons for factor: **TS**

**Comparison Diff of Means t Unadjusted P Critical Level Significant?**

CT vs. NT 376.800 3.718 <0.001 0.050 Yes

**Two Way Analysis of Variance** Friday, September 21, 2012, 6:00:45 AM

**Data source:** Nyoli-Babies in 2011 Notebook1

Balanced Design

Dependent Variable: MZ GY

**Normality Test (Shapiro-Wilk)** Passed (P = 0.549)

**Equal Variance Test:** Passed (P = 0.160)

**Source of Variation DF SS MS F P**

TS 1 13201666.667 13201666.667 38.246 <0.001

CS 1 37340.741 37340.741 0.108 0.743

TS x CS 1 99674.074 99674.074 0.289 0.592

Residual 92 31756518.519 345179.549

Total 95 45095200.000 474686.316

The difference in the mean values among the different levels of TS is greater than would be expected by chance after allowing for effects of differences in CS. There is a statistically significant difference (P = <0.001). To isolate which group(s) differ from the others use a multiple comparison procedure.

The difference in the mean values among the different levels of CS is not great enough to exclude the possibility that the difference is just due to random sampling variability after allowing for the effects of differences in TS. There is not a statistically significant difference (P = 0.743).

The effect of different levels of TS does not depend on what level of CS is present. There is not a statistically significant interaction between TS and CS. (P = 0.592)

Power of performed test with alpha = 0.0500: for TS : 1.000

Power of performed test with alpha = 0.0500: for CS : 0.0500

Power of performed test with alpha = 0.0500: for TS x CS : 0.0500

Least square means for TS :

**Group Mean**

CT 2029.167

NT 1287.500

Std Err of LS Mean = 84.801

Least square means for CS :

**Group Mean**

CM 1638.611

S-M 1678.056

Std Err of LS Mean = 84.801

Least square means for TS x CS :

**Group Mean**

CT x CM 1977.222

CT x S-M 2081.111

NT x CM 1300.000

NT x S-M 1275.000

Std Err of LS Mean = 119.927

**Two Way Analysis of Variance** Monday, May 05, 2014, 6:10:34 PM

**Data source:** Nyoli-Babies in 2013-ANALYSIS.JNB

Balanced Design

Dependent Variable: GYIELD

**Normality Test (Shapiro-Wilk)** Passed (P = 0.206)

**Equal Variance Test:** Passed (P = 0.490)

**Source of Variation DF SS MS F P**

TS 1 13791557.357 13791557.357 65.821 <0.001

CS 1 156816.683 156816.683 0.748 0.389

TS x CS 1 2268.520 2268.520 0.0108 0.917

Residual 92 19277000.107 209532.610

Total 95 33227642.666 349764.660

The difference in the mean values among the different levels of TS is greater than would be expected by chance after allowing for effects of differences in CS. There is a statistically significant difference (P = <0.001). To isolate which group(s) differ from the others use a multiple comparison procedure.

The difference in the mean values among the different levels of CS is not great enough to exclude the possibility that the difference is just due to random sampling variability after allowing for the effects of differences in TS. There is not a statistically significant difference (P = 0.389).

The effect of different levels of TS does not depend on what level of CS is present. There is not a statistically significant interaction between TS and CS. (P = 0.917)

Power of performed test with alpha = 0.0500: for TS : 1.000

Power of performed test with alpha = 0.0500: for CS : 0.0500

Power of performed test with alpha = 0.0500: for TS x CS : 0.0500

Least square means for TS :

**Group Mean**

CT 1919.167

NT 1161.111

Std Err of LS Mean = 66.070

Least square means for CS :

**Group Mean**

CM 1499.722

MZ-SB 1580.556

Std Err of LS Mean = 66.070

Least square means for TS x CS :

**Group Mean**

CT x CM 1873.889

CT x MZ-SB 1964.444

NT x CM 1125.556

NT x MZ-SB 1196.667

Std Err of LS Mean = 93.437
